# Analysis of the Effector Functions of Vδ2 γδ T Cells and NK Cells against Cholangiocarcinoma Cells

**DOI:** 10.3390/cells13161322

**Published:** 2024-08-08

**Authors:** Inthuon Kulma, Kesara Na-Bangchang, Andrea Carvallo Herrera, Ifeanyi Theodora Ndubuisi, Masashi Iwasaki, Hiromi Tomono, Craig T. Morita, Haruki Okamura, Hiroshi Mukae, Yoshimasa Tanaka

**Affiliations:** 1 Center for Medical Innovation, Nagasaki University, 1-7-1 Sakamoto, Nagasaki 852-8588, Japan; inthuorn.kulma@gmail.com (I.K.); carvalloandy98@gmail.com (A.C.H.); ifylove200@yahoo.com (I.T.N.); 2 Graduate Program in Bioclinical Sciences, Chulabhorn International College of Medicine, Thammasat University (Rangsit Campus), Pathum Thani 12121, Thailand; kesaratmu@yahoo.com; 3 Center for Innovation in Immunoregulative Technology and Therapeutics, Graduate School of Medicine, Kyoto University, Kyoto 606-8501, Japan; masashi.iwasaki@astellas.com; 4 Department of Respiratory Medicine, Graduate School of Biomedical Sciences, Nagasaki University, Nagasaki 852-8501, Japan; htomono@nagasaki-u.ac.jp (H.T.); hmukae@nagasaki-u.ac.jp (H.M.); 5 Department of Internal Medicine, University of Iowa, Iowa City, IA 52246, USA; craig-morita@uiowa.edu; 6 Laboratory of Tumor Immunology and Cell Therapy, Hyogo College of Medicine, Nishinomiya 663-8501, Japan; haruoka@hyo-med.ac.jp

**Keywords:** bisphosphonate, cancer immunotherapy, cholangiocarcinoma, γδ T cell, interleukin-18, natural killer cell

## Abstract

Cholangiocarcinoma (CCA) is a rare disease characterized by malignant cells derived from the epithelial cells of the biliary duct system. Despite extensive treatments, the prognosis for CCA remains poor, emphasizing the critical need for the development of novel treatments. Considerable attention has been directed towards innate immune effector cells, which can recognize tumor cells independently of the major histocompatibility complex, laying the foundation for the development of off-the-shelf drugs. In this study, we cultured innate immune cells obtained from the peripheral blood of healthy adults and conducted a comparative analysis of the effector functions against CCA cell lines by Vδ2 γδ T cells and NK cells. This analysis was performed using standard short- and long-term cytotoxicity assays, as well as ELISA for IFN-γ. Vδ2 γδ T cells demonstrated cytotoxicity and IFN-γ production in response to CCA cells in a TCR-dependent manner, particularly in the presence of tetrakis-pivaloyloxymethyl 2-(thiazole-2-ylamino)ethylidene-1,1-bisphosphonate, a bisphosphonate prodrug. In contrast, direct killing and antibody-dependent cellular cytotoxicity were relatively slow and weak. Conversely, NK cells displayed potent, direct cytotoxicity against CCA cells. In summary, both Vδ2 γδ T cells and NK cells show promise as innate immune effector cells for adoptive transfer therapy in the context of CCA.

## 1. Introduction

Cholangiocarcinoma (CCA), a type of bile duct cancer, is a malignant tumor originating from biliary epithelial cells [1]. CCA is classified into three subtypes based on its anatomical locations: intrahepatic CCA, perihilar CCA, and distal extrahepatic CCA [2]. The incidence of CCA varies geographically, with higher rates reported in Eastern countries compared to Western countries. Particularly noteworthy is the statistically significant prevalence rate in the northeastern region of Thailand [3,4,5]. In this region, the incidence rates of CCA are 85, 14.5, and 5.7 per 100,000 people in the northeast, north and central, and southern regions of Thailand, respectively. These variations are attributed in part to the differential distribution of risk factors among these regions [5].

Meta-analyses indicate that risk factors significantly associated with CCA, with lower limits for the 95% confidence interval of the adjusted odds ratio (AOR) ≥ 1.50, include old age, *Opisthorchis viverrini* infections, consumption of raw fish, family history of cancer, alcohol consumption, and praziquantel use [6]. Human liver fluke infections caused by *O. viverrini* pose a significant public health concern in Southeast Asia [7]. *O. viverrini* is prevalent in Thailand, the Lao People’s Democratic Republic, Cambodia, and central Vietnam [7]. The northeastern part of Thailand, where *O. viverrini* infections are widespread, exhibits a high incidence rate of CCA. *O. viverrini*-induced CCA results from chronic inflammation, potentially caused by mechanical irritation from the oral and ventral suckers of the fluke and metabolic products of the fluke. Additionally, certain studies suggest that parasite-specific immune responses may play a role in the development of CCA [8,9].

While surgical resection of the tumor is the recommended first-line treatment for CCA, only 25% of patients have resectable tumors at the time of diagnosis [10]. Due to considerable heterogeneity in clinical manifestations and a lack of specific diagnostic biomarkers for CCA, early-stage diagnosis remains a challenging and formidable task [11,12]. Chemotherapy is recommended to enhance the overall survival rate of unresectable or advanced-stage CCA patients [12]. Either gemcitabine alone or gemcitabine-based regimens are suggested for unresectable CCA [13]. The clinical efficacy of these drugs, however, remains unsatisfactory, raising concerns that chemotherapy might induce the emergence of multi-drug-resistant tumor cells. This could contribute to the recurrence of more formidable cases of CCA.

Immunotherapeutic treatments involving PD-1 immune checkpoint inhibitors and the adoptive transfer of immune effector cells have emerged for CCA in the past decade. A combination therapy utilizing anti-PD-1 monoclonal antibodies and a multiple kinase inhibitor targeting vascular endothelial growth factor receptors (EGFRs) has demonstrated efficacy in reducing lesion size in recurrent CCA patients [14]. This combined approach also shows promise in controlling bone metastases associated with CCA [14]. Despite these advancements, the prognosis for CCA remains notably poor compared to other types of tumors [15].

Recently, there has been significant attention directed towards the adoptive transfer of immune effector cells, including Vδ1 γδ T cells, Vδ2 γδ T cells, and natural killer (NK) cells, which play crucial roles in innate immune responses [16]. The majority of γδ T cells in adults express Vγ2Vδ2 (also known as Vγ9Vδ2)-bearing T cell receptors (TCRs) (hereafter referred to as Vδ2 γδ T cells). They recognize self isopentenyl diphosphate (IPP) and dimethylallyl diphosphate (DMAPP) in the mevalonate pathway, as well as foreign (*E*)-4-hydroxy-3-methylbut-2-enyl diphosphate (HMBPP) in the non-mevalonate pathway, also referred to as the 1-deoxy-**D**-xylulose 5-phosphate/2-*C*-methyl-**D**-erythritol 4-phosphate (DOXP/MEP) pathway in pathogenic microbes (Supplementary Scheme S1) [17,18,19]. Importantly, this recognition occurs in a butyrophilin (BTN) 2A1/3A1-dependent and major histocompatibility complex (MHC)-independent manner (Supplementary Scheme S2) [20,21]. When antigen-presenting cells or tumor cells are exposed to nitrogen-containing bisphosphonates (N-BPs), such as zoledronic acid and pamidronate, N-BPs inhibit farnesyl diphosphate synthase (FDPS) [18,22]. This inhibition leads to the intracellular accumulation of IPP and DMAPP, direct upstream metabolites of FDPS. IPP and DMAPP then bind to the B30.2 domain of BTN3A1 [23]. The interaction between B30.2 and IPP/DMAPP is somehow recognized by Vδ2 γδ T cells in the context of BTN2A1/3A1 [23]. In humans, NK cells are characterized by a CD3^−^CD56^+^ phenotype and demonstrate anti-tumor activity against various types of tumor cells [24]. While it has been reported that there are several subtypes of NK cells, the comprehensive elucidation of their phenotypic and functional aspects is still underway [25]. It is noteworthy that Vδ2 γδ T cells and NK cells express a variety of co-stimulatory molecules such as ICOS, and co-inhibitory molecules including PD-1 and NKG2A/CD94 [26,27,28,29,30,31]. This suggests that therapeutic efficacy could be modulated by enhancing or inhibiting these co-modulatory signals [32,33].

In this study, we expanded Vδ2 γδ T cells using tetrakis-pivaloyloxymethyl 2-(thiazole-2-ylamino)ethylidene-1,1-bisphosphonate (PTA), an N-BP prodrug, and interleukin-2 (IL-2). Additionally, NK cells were expanded in the presence of IL-2/IL-18 [34]. We then assessed the anti-tumor activity of Vδ2 γδ T cells and NK cells against CCA cell lines and explored the potential for the adoptive transfer of Vδ2 γδ T cells and NK cells.

## 2. Materials and Methods

### 2.1. Derivation of Vδ2 γδ T Cells

To expand Vδ2 γδ T cells, peripheral blood mononuclear cells (PBMCs) obtained from healthy adult donors after approval from the Institutional Review Board of Nagasaki University Hospital were stimulated with 1 μM PTA (Techno Suzuta Co., Ltd., Heiwa-machi, Nagasaki, Japan) and 100 IU/mL IL-2 (Kyowa Pharmaceutical Industries Co., Ltd., Kita-ku, Osaka, Japan) for 11 days. In this study, a total of 11 healthy donors were recruited (Supplementary Table S1). The expanded Vδ2 γδ T cells were then cryopreserved at −80 °C until used, as outlined in Supplementary Figure S1.

### 2.2. Flow Cytometric Analysis

PBMCs before and after expansion were analyzed for cell surface markers using flow cytometry, as described in Supplementary Figure S1. CCA cells were evaluated for the expression of EGFR and Her2. CCA cells were dispensed into the wells of a round-bottom 96-well plate at a cell concentration of 2 × 10^5^ cells/100 μL. The plate was then centrifuged at 600× *g* and 4 °C for 2 min. After removing the supernatants by flipping, the cell pellets were dispersed by vortexing and resuspended in 50 μL of phosphate-buffered saline (PBS)/2% fetal calf serum (FCS) containing 3 μL of biotin-conjugated anti-EGFR or Her2 mAb and green fluorescence protein (GFP)-conjugated biotin-binding protein. Following a 15 min incubation period on ice, 200 μL of PBS/2% FCS were added to the wells. The plate was centrifuged at 600× *g* and 4 °C for 2 min, and the supernatants were removed. After vortexing the plate, 200 μL of PBS/2% FCS were added to the wells. This process was repeated two more times, and the cells were finally resuspended in 200 μL of PBS/2% FCS and analyzed using a FACS Lyrics flow cytometer (Becton, Dickinson and Co., Franklin Lakes, NJ, USA).

### 2.3. Microscopic Analysis

Cell culture plates and flasks with cell suspensions were positioned under a microscope equipped with a 4× objective lens and a 10× eyepiece lens. Images were captured using cellSens software ver. 2.3 (Olympus Corp., Hachioji, Tokyo, Japan).

### 2.4. Maintenance of Human Cholangiocarcinoma Cell Lines

Eight human CCA cell lines were utilized in this study: HuCCT1 derived from the intrahepatic bile duct tree; TFK-1 from the extrahepatic bile duct; RBE from intrahepatic cholangiocarcinoma; TGBC1TKB and TGBC2TKB from gallbladder carcinoma that had metastasized to the lymph nodes; Mz-ChA-1 and Mz-ChA-2 from gallbladder adenocarcinoma metastases [35]; and Sk-ChA-1 from the malignant ascites of a patient with primary adenocarcinoma of the extrahepatic biliary tree [35]. The CCA cell lines were maintained as described in Supplementary Figure S2.

### 2.5. Time-Resolved Fluorescence-Based Short-Term Cellular Cytotoxicity Assay

Short-term γδ T cell-mediated cellular cytotoxicity was conducted to assess the killing of target CCA cells by Vδ2 γδ T cells and NK cells using a non-radioactive cellular cytotoxicity assay kit (Techno Suzuta Co., Ltd.) following the manufacturer’s protocol, as detailed in the Materials and Methods section of Supplementary Figure S2. To investigate the effect of PTA on short-term γδ T cell-mediated cellular cytotoxicity, CCA cells were treated with 0, 100, or 500 nM of PTA for 2 h before exposure to bis(butyryloxymethyl) 4′-(hydroxymethyl)-2,2′:6′,2″-terpyridine-6,6″-dicarboxylate (BM-HT, Techno Suzuta Co., Ltd.). For the analysis of the effects of anti-epidermal growth factor receptor (EGFR) mAb (Merck Biopharma Co., Ltd., Meguro-ku, Tokyo, Japan) and anti-Her2 mAb (Chugai Pharmaceutical Co., Ltd., Chuo-ku, Tokyo, Japan), CCA cells were treated with 0, 1, or 10 μg/mL of mAbs for 15 min before labeling with BM-HT. A time-resolved fluorescence-based assay was conducted at various effector-to-target (E/T) ratios: 0:1, 3.125:1, 6.25:1, 12.5:1, 25:1, 50:1, 100:1, and 200:1.

NK cell-mediated short-term cellular cytotoxicity was assessed using the same protocol as for Vδ2 γδ T cells, with E/T ratios set at 0:1, 1.25:1, 2.5:1, 5:1, 10:1, 20:1, 40:1, and 80:1. CCA cells were treated with 0, 0.01, or 0.1 μg/mL of anti-EGFR mAb. Time-resolved fluorescence was measured using a NIVO multi-plate reader (Revvity Inc., Yokohama, Kanagawa, Japan).

### 2.6. Luciferase-Based Long-Term Cellular Cytotoxicity Assay

Long-term γδ T cell-mediated cellular cytotoxicity was evaluated using a CellTiter-Glo^®^ Luminescent Cell Viability assay kit (Promega, Madison, WI, USA) following the manufacturer’s protocol, as detailed in Supplementary Figure S3. CCA cells were seeded into the wells of a 96-well plate (2 × 10^4^ cells/200 μL/well) and incubated at 37 °C with 5% CO_2_ overnight. After aspirating the culture supernatants, Vδ2 γδ T cells (200 μL each) were added to the wells at E/T ratios of 0:1, 1.56:1, 3.13:1, 6.25:1, 12.5:1, 25:1, 50:1, 100:1, and 200:1 in triplicate. Following a 72 h incubation period at 37 °C with 5% CO_2_, the wells were washed three times with 200 μL of RPMI1640 medium supplemented with 10% heat-inactivated FCS. Then, 100 μL of medium was added to each well. Subsequently, 100 μL of CellTiter-Glo Reagent was added to the wells, mixed well, and transferred to a Perkin Elmer OptiPlate 96 plate. Luminescence was measured using a NIVO multi-plate reader (Revvity Inc.).

To investigate the effect of PTA on γδ T cell-mediated long-term cellular cytotoxicity, CCA cells (2 × 10^4^ cells) were treated with various concentrations of PTA (0, 0.1, 1, 10, and 100 pM; 1, 10, and 100 nM; and 1 μM) at 37 °C with 5% CO_2_ for 2 h. Subsequently, the cells were challenged with 8 × 10^5^ cells/100 μL of Vδ2 γδ T cells at an E/T ratio of 40:1. For the analysis of the effect of anti-EGFR mAb (Merck Biopharma Co., Ltd., Meguro-ku, Tokyo, Japan), CCA cells (2 × 10^4^ cells) were treated with various concentrations of mAb (0, 128, and 640 pg/mL; 3.2, 16, 80, and 300 ng/mL; and 2 and 10 μg/mL) for 15 min and then challenged with 8 × 10^5^ cells/100 μL of Vδ2 γδ T cells at an E/T ratio of 40:1. After a 72 h incubation period at 37 °C with 5% CO_2_, the wells were washed three times with 200 μL of RPMI1640 medium supplemented with 10% heat-inactivated FCS. Next, 100 μL of medium was added to each well. Subsequently, 100 μL of CellTiter-Glo Reagent was added to the wells, mixed well, and transferred to a Perkin Elmer OptiPlate 96 plate. Luminescence was measured using a NIVO multi-plate reader (Revvity Inc.).

### 2.7. Enzyme-Linked Immunosorbent Assay for IFN-γ

To assess the secretion of IFN-γ from Vδ2 γδ T cells in response to PTA-pulsed CCA cells, a standard enzyme-linked immunosorbent assay (ELISA) was conducted following the procedure described in the Materials and Methods section of Supplementary Figure S3.

### 2.8. Derivation of NK Cells

For the expansion of NK cells, CD3^+^ cell-depleted PBMCs obtained from healthy adult donors were stimulated with 100 ng/mL IL-18 (Techno Suzuta Co., Ltd.) and 100 IU/mL IL-2 (Kyowa Pharmaceutical Industries Co., Ltd.) for 10 days. The expanded NK cells were then cryopreserved at −80 °C until used, following the procedure outlined in Supplementary Figure S6.

### 2.9. Statistical Analysis

The data are representative of three independent experiments and presented as the mean ± SD. Statistical significance was determined using a *t*-test, implemented in GraphPad Prism software version 8.4.3. A *p*-value < 0.05 was considered statistically significant.

## 3. Results

### 3.1. Vδ2 γδ T Cells Exhibit Cytotoxic Activity against CCA Cells

To prepare Vδ2 γδ T cells, PBMCs from healthy donors 1–4 (HD1–HD4) were stimulated with PTA/IL-2. Supplementary Figure S1A shows the generation of a large, highly homogeneous population of Vδ2 γδ T cells with a purity of 96% or greater, achieved without additional purification steps. Notably, cell clustering initiated on day 4 and reached its maximum on day 6 (Supplementary Figure S1B). By day 11, Vδ2 γδ T cells expressed γδ T cell receptors (TCRs), along with high levels of NKG2D and DNAM-1, both essential for innate immune cell effector functions.

Additionally, they demonstrated variable expression levels of CD16, an Fc receptor (Supplementary Figure S1C), and PD-1, NKG2A, and CD94, which are inhibitory receptors (Supplementary Figure S1D), depending on the individual. This prompts an intriguing exploration into whether Vδ2 γδ T cells exhibit TCR-mediated, NK receptor-mediated, and antibody-dependent cellular cytotoxicity against CCA cells.

**Figure 1:**
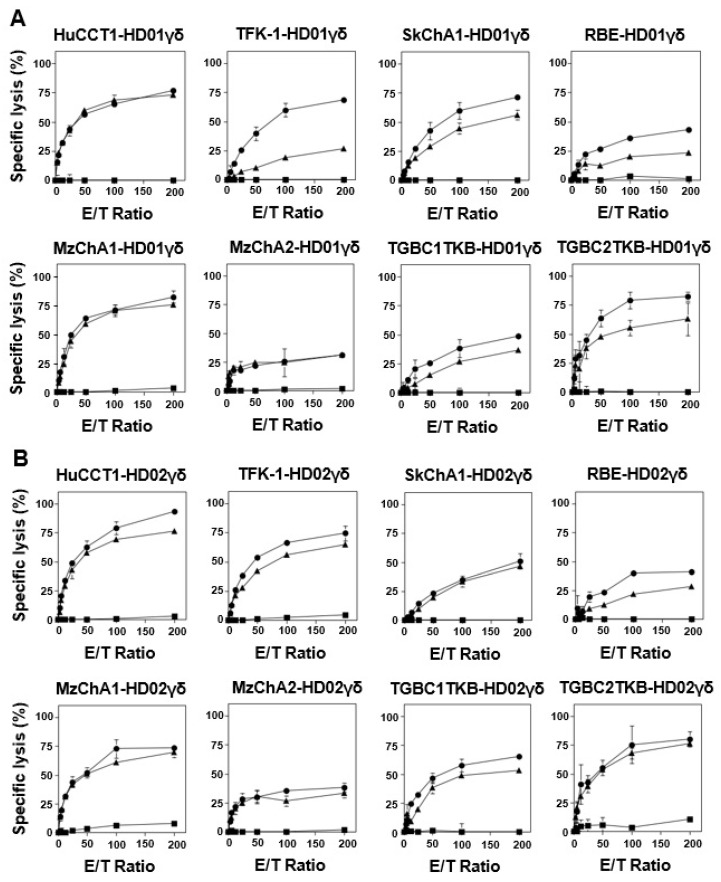
Short-term cellular cytotoxicity of Vδ2 γδ T cells against CCA cells. Comparison between TCR-dependent and independent cellular cytotoxicity exhibited by γδ cells derived from HD01, healthy donor 01 (**A**), and HD02, healthy donor 02 (**B**). After CCA cells were pretreated with 0 nM (■), 100 nM (▲), or 500 nM (•) of PTA, eliciting γδ TCR-mediated signaling, the unsensitized and PTA-sensitized CCA cells were challenged by a serial dilution of Vδ2 γδ T cells derived from HD01 or HD02 at E/T ratios of 0:1, 3.125:1, 6.25:1, 12.5:1, 25:1, 50:1, 100:1, and 200:1. The specific lysis (%) of CCA cells by Vδ2 γδ T cells within 1 h was plotted against E/T ratios.

In the initial phase of our study, we compared NK-like direct cytotoxicity and γδ TCR-mediated cytotoxicity of Vδ2 γδ T cells against CCA cells using a short-term cellular cytotoxicity assay system. Eight CCA cell lines—HuCCT1, TFK-1, SkChA1, RBE, MzChA1, MzChA2, TGBC1TKB, and TGBC2TKB—were employed as targets for immune effector cells. Upon direct challenge by HD01 and HD02 Vδ2 γδ T cells, minimal cytotoxic activity was observed within the first hour (filled squares in Figure 1). However, when CCA cells were pretreated with PTA, E/T ratio-dependent killing was observed across all eight cell lines (filled triangles for 100 nM PTA and filled circles for 500 nM PTA in Figure 1). Notably, HuCCT1 and TGBC2TKB exhibited relatively higher sensitivity to PTA pretreatment plus γδ T cell challenge compared to other CCA cell lines, especially MzChA2. For example, specific lysis by HD01 γδ T cells at an E/T ratio of 100:1 in the presence of PTA was 64.9 ± 1.0% for HuCCT1, 79.1 ± 7.1% for TGBC2TKB, and 25.7 ± 1.8% for MzChA2. In contrast, in the absence of PTA, it was 0 ± 3.1% for HuCCT1, 0 ± 1.9% for TGBC2TKB, and 1.5 ± 2.5% for MzChA2, respectively.

To investigate whether the observed sensitivity of CCA cell lines to PTA pulsing plus γδ T cell challenge extends to other donors, we expanded Vδ2 γδ T cells from HD05 and HD06 and conducted the same killing assay. Supplementary Figure S2A,B illustrates that the sensitivity of HuCCT1 and TGBC2TKB to PTA pretreatment plus γδ T cell challenge remained higher than that of other CCA cell lines, particularly MzChA2. Remarkably, approximately 50% of PTA-pulsed HuCCT1 and TGBC2TKB cells were killed by Vδ2 γδ T cells at an E/T ratio of 50:1 within 1 h. When the specific lysis results from the four healthy donors were combined and statistically analyzed, it was confirmed that PTA-sensitized HuCCT1 and TGB2TKB were more susceptible to the cytotoxicity exhibited by Vδ2 γδ T cells compared to similarly pretreated MzChA2. This suggests that the cytotoxicity of Vδ2 γδ T cells against CCA cells might depend on certain factors within the CCA cells (Supplementary Figure S2C). We then analyzed the expression of PD-L1 and HLA-E, ligands for the PD-1 and NKG2A/CD94 inhibitory receptors, respectively. As shown in Supplementary Figure S2D,E, these ligands were differentially expressed on CCA cell lines, and no clear correlation to cytotoxicity was observed.

### 3.2. Vδ2 γδ T Cells Require an Extended Timeframe to Exhibit Cytotoxicity against CCA Cells

**Figure 2:**
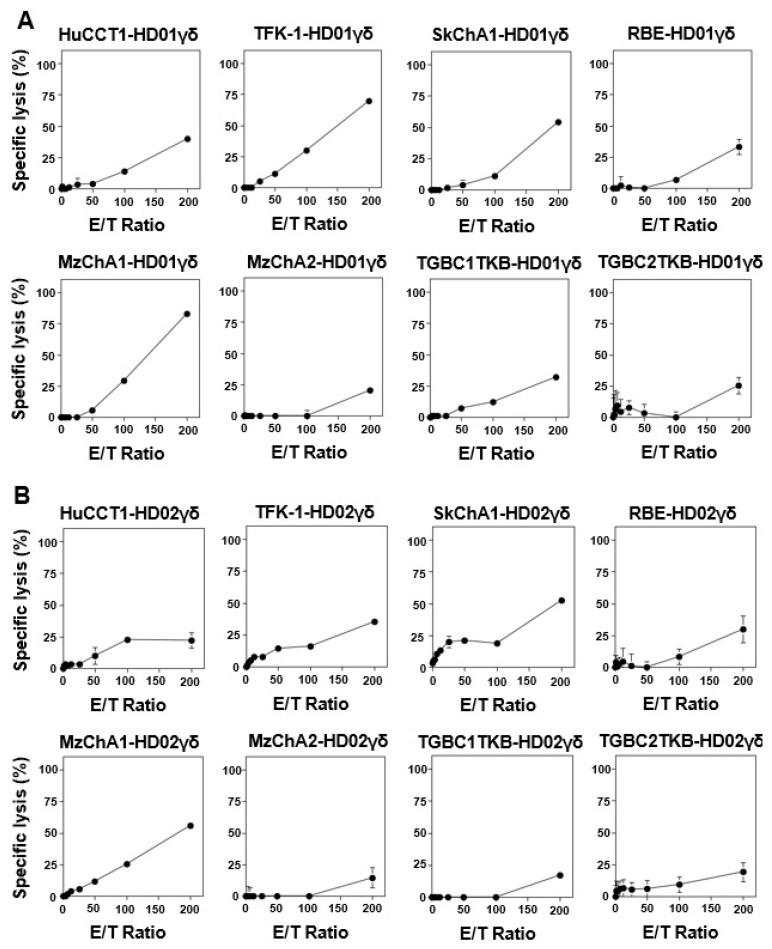
Long-term cytotoxic activity exhibited by Vδ2 γδ T cells against CCA cell lines. (**A**) γδ cells derived from a healthy donor, HD01. (**B**) γδ cells derived from a healthy donor, HD02. CCA cells were seeded into the wells of a 96-well plate and incubated at 37 °C with 5% CO_2_ overnight. After aspirating the culture supernatants, Vδ2 γδ T cells were added to the wells at E/T ratios of 0:1, 1.56:1, 3.13:1, 6.25:1, 12.5:1, 25:1, 50:1, 100:1, and 200:1 in triplicate. Following a 72 h incubation period, CellTiter-Glo Reagent was added to the wells and luminescence was measured.

Given that CCA cells exhibited little to no susceptibility to γδ T cell-mediated cytotoxicity within 1 h, even at an E/T ratio of 200:1 in the absence of PTA, we explored whether CCA cells were intrinsically resistant to γδ T cell cytotoxicity or if extended exposure could induce cytotoxicity. Upon incubating CCA cells with Vδ2 γδ T cells for 72 h, E/T ratio-dependent cellular cytotoxicity was observed (Figure 2). This suggests that Vδ2 γδ T cells require an extended duration to effectively kill CCA cells in the absence of additional stimuli that mediate TCR signaling and/or Fc receptor signaling. In this long-term cytotoxicity assay, MzChA1 displayed the highest sensitivity among the cell lines, followed by TFK-1, SkChA1, and HuCCT1, with MzChA2 being the least sensitive among those examined in this study. Notably, the order of sensitivity to Vδ2 γδ T cells in the long-term assay system slightly differed from the order in the short-term assay system. This discrepancy might be attributed to the dependence of cellular cytotoxicity on TCR signaling or other factors. However, it is worth mentioning that the order of sensitivity of CCA cell lines to Vδ2 γδ T cells remained consistent across the healthy donors.

### 3.3. TCR Signaling Facilitates the Effector Functions of Vδ2 γδ T Cells against CCA

Subsequently, we examined the effect of PTA on the long-term cellular cytotoxicity of Vδ2 γδ T cells against CCA cell lines. The assay was conducted with an E/T ratio of 40:1, ensuring specific lysis (%) remained below 30%, allowing the detection of the effect of TCR signaling on γδ T cell cytotoxicity against CCA cell lines after a 72 h incubation period. As illustrated in Supplementary Figure S3A, robust cellular cytotoxicity was evident when CCA cell lines were pre-treated with 100 nM or 1 μM PTA before γδ T cell challenge. Notably, the PTA-mediated enhancement of cellular cytotoxicity was consistently observed across all CCA cell lines.

Upon assessing IFN-γ in the culture supernatants of Vδ2 γδ T cells co-incubated with 1 μM PTA-pretreated CCA cell lines, it was observed that PTA significantly induced the secretion of IFN-γ from the Vδ2 γδ T cells. In contrast, Vδ2 γδ T cells exhibited varying degrees of IFN-γ secretion in response to 100 nM PTA-pretreated CCA, indicating that the specific lysis of CCA by Vδ2 γδ T cells is more sensitive than the production of IFN-γ by Vδ2 γδ T cells. For example, specific lysis was nearly 100% when 100 nM PTA-pretreated SkChA1 was challenged by Vδ2 γδ T cells for 72 h, whereas IFN-γ secretion was only marginal in the same culture (Supplementary Figure S3B). Thus, it appears that IFN-γ secretion and cytotoxicity are not parallel events and depend on the strength of TCR signaling. In addition, significant levels of degranulation and IFN-γ production were observed in Vδ2 γδ T cells when exposed to PTA-sensitized CCA cells through a CD107a-based degranulation assay and a flow cytometry-based intracellular IFN-γ staining assay, demonstrating that Vδ2 γδ T cells specifically recognized PTA-sensitized CCA cells (Supplementary Figure S3C,D).

### 3.4. CD16-Mediated Signaling Facilitates the Effector Functions of Vδ2 γδ T Cells against CCA

**Figure 3:**
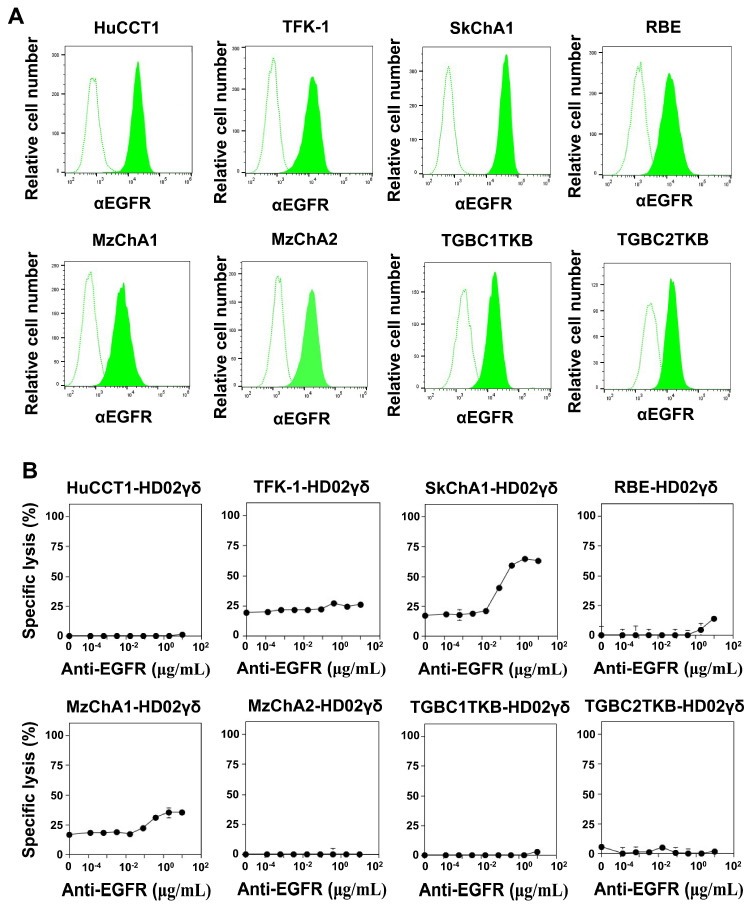
CD16-mediated cytotoxicity against CCA cells by Vδ2 γδ T cells. (**A**) Expression of EGFR on CCA cells. CCA cells were dispensed into the wells of a round-bottom 96-well plate, which was centrifuged. The cells were resuspended in PBS/2% FCS, supplemented with 3 μL of biotin-conjugated anti-EGFR mAb (filled histograms) or control Ab (unfilled histograms), followed by GFP-conjugated biotin-binding protein solution. Following a 15 min incubation period on ice, the cells were washed with PBS/2% FCS and examined for the expression of EGFR. (**B**) Effect of anti-EGFR mAb on the cytotoxic activity of Vδ2 γδ T cells against CCA cell lines in a long-time cytotoxicity assay. CCA cells were treated with various concentrations of anti-EGFR mAb (0, 128, and 640 pg/mL; 3.2, 16, 80, and 300 ng/mL; and 2 and 10 μg/mL) for 15 min and then challenged with Vδ2 γδ T cells at an E/T ratio of 40:1. After a 72 h incubation period, CellTiter-Glo Reagent was added to the wells and luminescence was measured.

To determine whether Vδ2 γδ T cells exhibit antibody-dependent cellular cytotoxicity (ADCC), CCA cell lines were analyzed for the expression of EGFR, an epidermal growth factor receptor. As shown in Figure 3A, all CCA cell lines expressed EGFR, with SkChA1 exhibiting the highest level. Vδ2 γδ T cells express CD16, an Fc receptor that recognizes the Fc region of antibodies bound to target cells, in varying degrees depending on the donor, with HD02 showing the highest level of CD16 expression. When CCA cell lines were treated with either 1 or 10 μg/mL anti-EGFR mAb and then challenged by HD02 Vδ2 γδ T cells, the anti-EGFR mAb-mediated ADCC was only marginal in the short-term assay system using a time-resolved fluorescence-based assay (Supplementary Figure S4).

When utilizing the luciferase-based long-term assay system, a relatively high level of anti-EGFR mAb-mediated antibody-dependent cellular cytotoxicity (ADCC) was observed for SkChA1. In contrast, Vδ2 γδ T cells exhibited only moderate to marginal levels of ADCC against other CCA cell lines (Figure 3B). Overall, while Vδ2 γδ T cells show potential for ADCC, CCA cell lines appear to be relatively resistant to the ADCC exerted by Vδ2 γδ T cells.

To further explore the ADCC exhibited by Vδ2 γδ T cells, the expression of Her2 in CCA cell lines was examined. As depicted in Supplementary Figure S5A, the CCA cell lines expressed only marginal levels of Her2. Furthermore, the ADCC mediated by Vδ2 γδ T cells using anti-Her2 mAb was also found to be marginal, confirming that the ADCC exhibited by Vδ2 γδ T cells was not significant for CCA cell lines (Supplementary Figure S5B).

### 3.5. IL2/IL-18-Induced NK Cells Exhibited Potent Cellular Cytotoxicity against CCA Cells

We next examined the effector functions of NK cells, another representative innate immune cell population, against CCA cells. After incubating CD3^+^ cell-depleted PBMCs (HD08-HD11) with IL-2/IL-18 for 10 days, the proportions of CD3^−^CD56^+^ NK cells greatly exceeded 90%, as shown in Supplementary Figure S6A. The cells began to form clusters from day 4 to 5 and proliferated thereafter (Supplementary Figure S6B). Then, the cells were harvested and examined for cell surface markers on day 10. As illustrated in Supplementary Figure S6C, CD3^−^CD56^+^ NK cells expressed high levels of NKG2D and DNAM-1, representative innate immune effector molecules, and CD16, an Fc receptor involved in ADCC. In addition, they exhibited high expression levels of CD86 and HLA-DQ, which are typically expressed on antigen-presenting cells such as macrophages and dendritic cells. Regarding inhibitory receptors, they expressed only a marginal level of PD-1, whereas a significant level of NKG2A/CD94 expression was observed, as shown in Supplementary Figure S6D.

**Figure 4:**
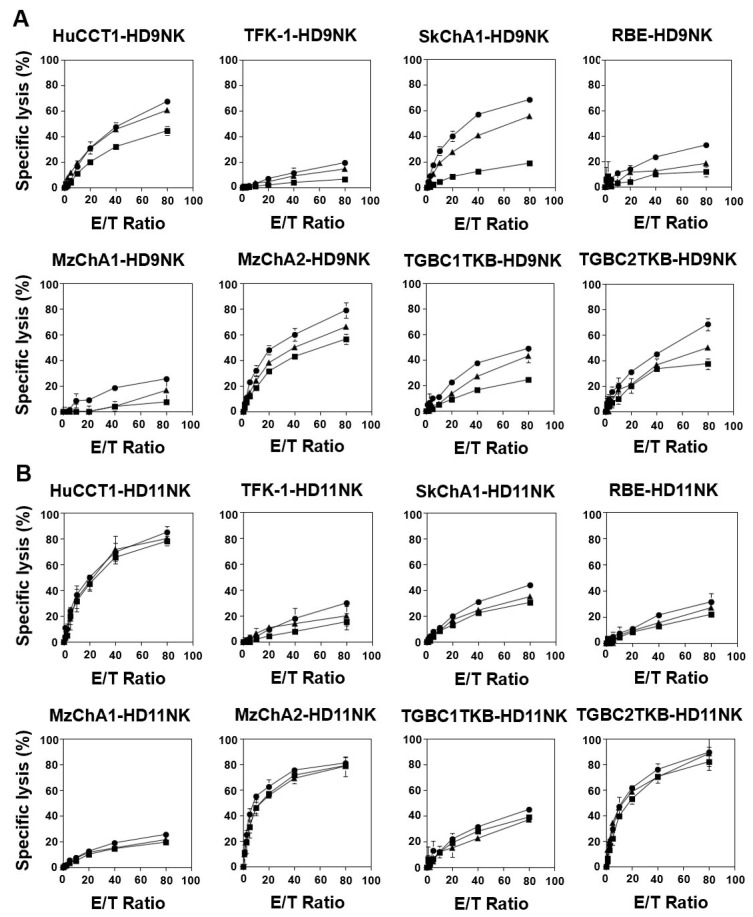
Cytotoxicity of IL-2/IL-18-expanded NK cells against CCA cell lines. CCA cells were pretreated with 0 (■), 0.01 (▲), or 0.1 μg/mL (●) anti-EGFR mAb and subsequently challenged by NK cells derived from: (**A**) Healthy donor HD09; (**B**) Healthy donor HD11. NK cell-mediated short-term ADCC was assessed with E/T ratios set at 0:1, 1.25:1, 2.5:1, 5:1, 10:1, 20:1, 40:1, and 80:1. Then, time-resolved fluorescence was measured.

Subsequently, we determined the effector functions of IL-2/IL-18-induced NK cells against CCA cell lines using an Eu-based short-term cellular cytotoxicity assay system. As shown in Figure 4A, HD09 NK cells exhibited direct cytotoxicity against all eight CCA cell lines. In particular, approximately 40% specific lysis or greater was observed in HuCCT1, MzChA2, and TGBC2TKB within 1 h at an E/T ratio of 80:1. When anti-EGFR mAb was included in this assay system, markedly higher levels of cytotoxicity were observed in all CCA cell lines. For example, specific lysis by HD09 NK cells at an E/T ratio of 80:1 in the presence of 0.1 μg/mL anti-EGFR mAb was 67.5 ± 2.8% for HuCCT1, 79.2 ± 6.0% for MzChA2, and 68.5 ± 4.8% for TGBC2TKB. In contrast, in the absence of mAb, it was 44.6 ± 3.5% for HuCCT1, 56.7 ± 3.8% for MzChA2, and 37.5 ± 4.3% for TGBC2TKB, respectively.

To examine whether this is the case for another donor, HD11 NK cells were subjected to the same assay systems, revealing that HuCCT1, MzChA2, and TGBC2TKB were highly sensitive to the direct cytotoxicity by HD11 NK cells, and the add-on effect of anti-EGFR mAb was observed in all CCA cell lines to different degrees (Figure 4B). It is noteworthy that the additive effect of the mAbs tended to be small when natural killer activity was relatively high. For example, specific lysis by HD09 NK cells at an E/T ratio of 80:1 increased by 22.5% (from 56.7 ± 3.8% to 79.2 ± 6.0%) with the addition of 0.1 μg/mL anti-EGFR mAb, whereas the increase for HD11 was 7.0% (from 78.4 ± 3.6% to 85.4 ± 4.5%). This is probably because cellular cytotoxicity was nearly saturated when natural killing activity was relatively high, as in the case of HD11.

## 4. Discussion

CCA is an elusive yet rare disease, though it constitutes a significant public health concern in certain regions of East and Southeast Asian countries [5]. Given the constrained effectiveness of standard therapies, the need to devise innovative therapeutic approaches for CCA is paramount [36]. In this study, we investigated the therapeutic capabilities of Vδ2 γδ T cells and NK cells. These innate immune effector cells, unrestricted by MHC class I/II molecules, offer the potential for developing readily available drugs that harness their potential.

Regarding the expansion of these innate immune cells, Vδ2 γδ T cells constitute approximately 2–4% of peripheral blood lymphocytes [37,38,39] and demonstrate substantial proliferation when stimulated with PTA followed by culture in the presence of IL-2. Typically, the expansion rate of Vδ2 γδ T cells ranges from 1000 to 3000-fold after 11 days of culture. In contrast, NK cells make up 10 to 30% of peripheral blood lymphocytes, yet their expansion rate within 10 days is only several-fold to several dozen-fold, even after culture with IL-2/IL-18. In this context, Vδ2 γδ T cells exhibit superiority over NK cells.

When CCA cells were exposed to Vδ2 γδ T cells, direct cellular cytotoxicity was only marginal within the first hour. However, extending the culture period to up to 72 h revealed a significant increase in cellular cytotoxicity. This suggests that Vδ2 γδ T cells require a relatively extended timeframe to exhibit cytotoxicity against CCA cells, underscoring the need for a large number of highly purified Vδ2 γδ T cells to prolong the contact time between CCA cells and Vδ2 γδ T cells for effective elimination. Furthermore, it is advisable to utilize allogeneic Vδ2 γδ T cells derived from healthy donors in the development of γδ T cell-based adoptive cancer immunotherapy, as expanding innate immune effector cells from cancer patients is generally challenging [40,41].

When PTA was introduced into this system, a notably elevated level of cellular cytotoxicity against CCA was observed within 1 h, highlighting the significance of TCR-mediated signaling in the short-term cellular cytotoxicity of Vδ2 γδ T cells. A crucial consideration revolves around the delivery of PTA to tumor cells. Given PTA’s high hydrophobicity, it can permeate the cell membranes of various cell types. Therefore, it becomes imperative to devise a drug delivery system that effectively targets CCA cells.

As Vδ2 γδ T cells express CD16 to varying degrees depending on the donor, the assessment of ADCC activity was conducted using Vδ2 γδ T cells expressing a high level of CD16. According to this study, ADCC activity against CCA was not significant, suggesting that, at present, the only viable strategy is to rely on direct and prolonged cellular cytotoxicity against CCA cells. To implement this approach successfully, it is crucial to generate a large number of highly purified Vδ2 γδ T cells. Consequently, our expansion system utilizing PTA and IL-2 emerges as an ideal method for preparing Vδ2 γδ T cells for adoptive transfer in both autologous and allogeneic settings. In addition, it appears essential to assess the expression level of CD16 when developing adoptive transfer of Vδ2 γδ T cells alongside monoclonal antibodies, given that only a limited proportion of donors express a high level of CD16 on Vδ2 γδ T cells.

In terms of the cellular cytotoxicity exhibited by NK cells, despite a moderate expansion rate, direct killing against CCA cells is notably high even in a short-term assay system. Furthermore, NK cells exhibit explicit ADCC activity, indicating their remarkable potential as effector cells against CCA if we can enhance NK cell expansion more efficiently ex vivo. In our expansion system, the combination of IL-2/IL-18 achieves a relatively high expansion rate. Notably, IL-18 enhances the expression of CD86 and HLA-DQ, known to be present on antigen-presenting cells such as macrophages and dendritic cells. Since IL-2/IL-18-induced NK cells both kill CCA cells and express markers associated with antigen-presenting cells, it is plausible that they could serve as antigen-presenting cells, presenting CCA-derived antigens to both CD4^+^ αβ T cells and CD8^+^ αβ T cells. If this holds true, IL-2/IL-18-induced NK cells function as a bridge between innate immunity and adaptive immunity, challenging the conventional view of NK cells solely as innate immune effector cells that naturally eliminate tumor cells. In this context, IL-2/IL-18-induced NK cells outperform Vδ2 γδ T cells in their effector functions, particularly in cellular cytotoxicity against CCA cells and their bridging functions to adaptive immunity.

We previously reported that Vδ2 γδ T cells exhibited high levels of cellular cytotoxicity against HTLV-1-infected cells even in the absence of PTA [42]. However, CCA cells were intrinsically resistant to Vδ2 γδ T cells under conditions without TCR triggering, aligning with previous findings that mesothelioma cells also show resistance to Vδ2 γδ T cells without TCR triggering [43]. In this context, the pattern of CCA recognition by Vδ2 γδ T cells resembles that of mesothelioma cells rather than HTLV-1 cells.

It has been demonstrated that Vδ2 γδ T cells efficiently recognize and regulate *Herpes simplex* virus infections [44], malaria parasite infections [45,46,47,48], and mycobacteria infections [49,50,51,52] in the absence of external antigen addition or TCR triggering. This suggests that Vδ2 γδ T cells are inherently linked to infection immunity, although the precise mechanism remains incompletely elucidated. One possibility is that pathogenic microbe infections somehow alter the mevalonate pathway, leading to an increase in intracellular levels of IPP and DMAPP in infected cells. This, in turn, stimulates Vδ2 γδ T cells in a TCR- and BTN2A1/3A1-dependent manner [23].

In contrast, the recognition of solid tumor-derived cell lines by Vδ2 γδ T cells is inherently different from that of pathogenic microbe-infected cells. The solid tumor-derived cell lines generally do not stimulate Vδ2 γδ T cells via TCR. Instead, Vδ2 γδ T cells seem to recognize solid tumor-derived tumor cells in an NK cell receptor-dependent manner. However, the NK-like killing of CCA cells by Vδ2 γδ T cells is notably slower compared to that by NK cells. This is likely due to the fact that NK cells express a broader spectrum of NK receptors and death ligands than Vδ2 γδ T cells. Moreover, NK cell-mediated cellular cytotoxicity is more intricately regulated by numerous receptors and death ligands than the NK-like killing exhibited by Vδ2 γδ T cells.

Previous studies have demonstrated that combining autologous NK cells, Vδ2 γδ T cells, and cytokine-induced killer (CIK) cells in therapy with radiofrequency ablation leads to higher progression-free survival (PFS) compared to radiofrequency ablation therapy alone in patients with hepatocellular carcinoma [53]. Additionally, adoptive transfer of allogeneic γδ T cell treatments has been shown to extend overall survival in various cancer patients [40,41]. The administration of allogeneic Vδ2 γδ T cells to a metastatic CCA patient resulted in no adverse effects and was found to regulate peripheral immune cells, such as αβ T cells and NK cells, by increasing functional CD3^+^CD4^+^CD28^+^ helper T cells, CD3^+^CD8^+^CD28^+^ killer T cells, and CD3^−^CD56^+^ NK cells. These increases were accompanied by a decrease in exhausted and aged CD4^+^ and CD8^+^ T cells, as well as a reduction in the size of lymph nodes following the adoptive transfer of Vδ2 γδ T cells. Furthermore, tumor markers (AFP and CA-199) were maintained at low levels during treatment. Similarly, adoptive allogeneic γδ T cell therapy in late-stage lung and liver cancer patients improved the frequencies of immune cells such as CD4^+^, CD8^+^, and Vδ2 γδ T cells [40,41].

Finally, it has become evident that Vδ2 γδ T cells and NK cells do not exert cytotoxic effects uniformly across all CCA cells, showing a bias in cytotoxicity. For example, Vδ2 γδ T cells exhibit high activity against HuCCT1, MzChA1, and TGBC2TKB but show relatively lower activity against RBE and MzChA2. Similarly, NK cells demonstrate high activity against HuCCT1, MzChA2, and TGBC2TKB but not against TFK-1, RBE, and MzChA1. This phenomenon is not specific to individual donors but is observed consistently across nearly all donors, suggesting that it is unlikely due to differences in MHC. In addition, there are CCA cells that are commonly more susceptible or more resistant to both Vδ2 γδ T cells and NK cells, while others show opposite susceptibilities. Considering these observations, it is possible that γδ T cell infusion therapy may be effective for some CCA patients, while NK cell therapy may be effective for others. This underscores the necessity of identifying biomarkers to distinguish between these cases. Alternatively, when culturing peripheral blood, it may be clinically feasible to use a combination of PTA/IL-18/IL-2 to simultaneously expand both Vδ2 γδ T cells and NK cells, potentially leading to the development of combined γδ T cell and NK cell therapy. In fact, it was recently demonstrated that Vδ2 γδ T cells exhibit potent cytotoxic activity against NK cell-resistant Huh-7 hepatocellular carcinoma cells, indicating that Vδ2 γδ T cells and NK cells may cooperate in eliminating hepatocellular carcinoma cells [54,55].

## 5. Conclusions

Both Vδ2 γδ T cells and NK cells exhibit promising potential as alternative therapeutic modalities for CCA, and the findings from the present study could pave the way for the development of a feasible adoptive transfer therapy.

## 6. Patents

Y.T. is a co-inventor of JP2014-257451 (on the method for expanding Vδ2 γδ T cells using PTA) and JP2014-73475 (on the method for a nonradioactive cellular cytotoxicity assay).

## Data Availability

The raw data supporting the conclusions of this article will be made available by the authors, without undue reservation.

## Supplementary Materials

The following supporting information can be downloaded at: https://www.mdpi.com/article/10.3390/cells13161322/s1; Supplementary Figure S1: Expansion of Vδ2 γδ T cells by PTA/IL-2 [56]; Supplementary Figure S2: Cytotoxicity displayed by Vδ2 γδ T cells against CCA cell lines pretreated with 0 (■), 100 (▲), or 500 nM (●) of PTA; Supplementary Figure S3. Effect of PTA on the effector functions of Vδ2 γδ T cells; Supplementary Figure S4. Effect of anti-EGFR mAb on CD16-mediated cytotoxicity by Vδ2 γδ T cells against CCA cells in short-term cellular cytotoxicity assays; Supplementary Figure S5. CD16-mediated cytotoxicity against CCA cells by Vδ2 γδ T cells; Supplementary Figure S6. Expansion of NK cells by IL-2/IL-18; Scheme S1. Human and microbial pathways for the biosynthesis of isoprenoid metabolites; Scheme S2. Distinct mechanisms govern the recognition of antigens by αβ T cells and Vδ2 γδ T cells; Table S1. Healthy donors’ characteristics.

## References

[1] Wattanawongdon W., Hahnvajanawong C., Namwat N., Kanchanawat S., Boonmars T., Jearanaikoon P., Leelayuwat C., Techasen A., Seubwai W. (2015). Establishment and characterization of gemcitabine-resistant human cholangiocarcinoma cell lines with multidrug resistance and enhanced invasiveness. Int. J. Oncol..

[2] Tepsiri N., Chaturat L., Sripa B., Namwat W., Wongkham S., Bhudhisawasdi V., Tassaneeyakul W. (2005). Drug sensitivity and drug resistance profiles of human intrahepatic cholangiocarcinoma cell lines. World J. Gastroenterol..

[3] Banales J.M., Cardinale V., Carpino G., Marzioni M., Andersen J.B., Invernizzi P., Lind G.E., Folseraas T., Forbes S.J., Fouassier L. (2016). Expert consensus document: Cholangiocarcinoma: Current knowledge and future perspectives consensus statement from the European Network for the Study of Cholangiocarcinoma (ENS-CCA). Nat. Rev. Gastroenterol. Hepatol..

[4] Vatanasapt V., Martin N., Sriplung H., Chindavijak K., Sontipong S., Sriamporn H., Parkin D.M., Ferlay J. (1995). Cancer incidence in Thailand, 1988–1991. Cancer Epidemiol. Biomarkers Prev..

[5] Khan S.A., Tavolari S., Brandi G. (2019). Cholangiocarcinoma: Epidemiology and risk factors. Liver Int..

[6] Kamsa-ard S., Luvira V., Suwanrungruang K., Vatanasapt P., Wiangnon S. (2018). Risk factors for cholangiocarcinoma in Thailand: A systematic review and meta-analysis. Asian Pac. J. Cancer Prev..

[7] Sripa B., Pairojkul C. (2008). Cholangiocarcinoma: Lessons from Thailand. Curr. Opin. Gastroenterol..

[8] Flavell D.J., Flavell S.U. (1986). Opisthorchis viverrini: Pathogenesis of infection in immunodeprived hamsters. Parasite Immunol..

[9] Haswell-Elkins M.R., Sithithaworn P., Mairiang E., Elkins D.B., Wongratanacheewin S., Kaewkes S., Mairiang P. (1991). Immune responsiveness and parasite-specific antibody levels in human hepatobiliary disease associated with Opisthorchis viverrini infection. Clin. Exp. Immunol..

[10] Valverde A., Bonhomme N., Farges O., Sauvanet A., Flejou J.F., Belghiti J. (1999). Resection of intrahepatic cholangiocarcinoma: A Western experience. J. Hepatobiliary Pancreat. Surg..

[11] Doherty B., Nambudiri V.E., Palmer W.C. (2017). Update on the diagnosis and treatment of cholangiocarcinoma. Curr. Gastroenterol. Rep..

[12] Malaguarnera G., Paladina I., Giordano M., Malaguarnera M., Bertino G., Berretta M. (2013). Serum markers of intrahepatic cholangiocarcinoma. Dis. Markers.

[13] Skipworth J.R., Olde Damink S.W., Imber C., Bridgewater J., Pereira S.P., Malagó M. (2011). Review article: Surgical, neo-adjuvant and adjuvant management strategies in biliary tract cancer. Aliment. Pharmacol. Ther..

[14] Chen W.X., Li G.X., Hu Z.N., Zhu P., Zhang B.X., Ding Z.Y. (2019). Significant response to anti-PD-1 based immunotherapy plus lenvatinib for recurrent intrahepatic cholangiocarcinoma with bone metastasis: A case report and literature review. Medicine.

[15] Banales J.M., Marin J.J.G., Lamarca A., Rodrigues P.M., Khan S.A., Roberts L.R., Cardinale V., Carpino G., Andersen J.B., Braconi C. (2020). Cholangiocarcinoma 2020: The next horizon in mechanisms and management. Nat. Rev. Gastroenterol. Hepatol..

[16] Gao Y., Yang W., Pan M., Scully E., Girardi M., Augenlicht L.H., Craft J., Yin Z. (2003). γδ T cells provide an early source of interferon gamma in tumor immunity. J. Exp. Med..

[17] Hintz M., Reichenberg A., Altincicek B., Bahr U., Gschwind R.M., Kollas A.K., Beck E., Wiesner J., Eberl M., Jomaa H. (2001). Identification of (*E*)-4-hydroxy-3-methyl-but-2-enyl pyrophosphate as a major activator for human gammadelta T cells in Escherichia coli. FEBS Lett..

[18] Gober H.J., Kistowska M., Angman L., Jenö P., Mori L., De Libero G. (2003). Human T cell receptor γδ cells recognize endogenous mevalonate metabolites in tumor cells. J. Exp. Med..

[19] Chen Z.W. (2013). Multifunctional immune responses of HMBPP-specific Vγ2Vδ2 T cells in *M. tuberculosis* and other infections. Cell. Mol. Immunol..

[20] Harly C., Guillaume Y., Nedellec S., Peigné C.M., Mönkkönen H., Mönkkönen J., Li J., Kuball J., Adams E.J., Netzer S. (2012). Key implication of CD277/butyrophilin-3 (BTN3A) in cellular stress sensing by a major human γδ T-cell subset. Blood.

[21] Sandstrom A., Peigné C.M., Léger A., Crooks J.E., Konczak F., Gesnel M.C., Breathnach R., Bonneville M., Scotet E., Adams E.J. (2014). The intracellular B30.2 domain of butyrophilin 3A1 binds phosphoantigens to mediate activation of human Vγ9Vδ2 T cells. Immunity.

[22] Dunford J.E. (2010). Molecular targets of the nitrogen containing bisphosphonates: The molecular pharmacology of prenyl synthase inhibition. Curr. Pharm. Des..

[23] Rigau M., Ostrouska S., Fulford T.S., Johnson D.N., Woods K., Ruan Z., McWilliam H.E.G., Hudson C., Tutuka C., Wheatley A.K. (2020). Butyrophilin 2A1 is essential for phosphoantigen reactivity by γδ T cells. Science.

[24] Levy E.M., Roberti M.P., Mordoh J. (2011). Natural killer cells in human cancer: From biological functions to clinical applications. J. Biomed. Biotechnol..

[25] Freud A.G., Mundy-Bosse B.L., Yu J., Caligiuri M.A. (2017). The broad spectrum of human natural killer cell diversity. Immunity.

[26] Fattori S., Gorvel L., Granjeaud S., Rochigneux P., Rouvière M.S., Ben Amara A., Boucherit N., Paul M., Dauplat M.M., Thomassin-Piana J. (2021). Quantification of immune variables from liquid biopsy in breast cancer patients links Vδ2. Cancers.

[27] Cazzetta V., Bruni E., Terzoli S., Carenza C., Franzese S., Piazza R., Marzano P., Donadon M., Torzilli G., Cimino M. (2021). NKG2A expression identifies a subset of human Vδ2 T cells exerting the highest antitumor effector functions. Cell Rep..

[28] Iwasaki M., Tanaka Y., Kobayashi H., Murata-Hirai K., Miyabe H., Sugie T., Toi M., Minato N. (2011). Expression and function of PD-1 in human γδ T cells that recognize phosphoantigens. Eur. J. Immunol..

[29] Ge M.R., Yang C.L., Li T., Du T., Zhang P., Li X.L., Dou Y.C., Duan R.S. (2023). Circulating CXCR5^+^ natural killer cells are expanded in patients with myasthenia gravis. Clin. Transl. Immunol..

[30] Kaulfuss M., Mietz J., Fabri A., Vom Berg J., Münz C., Chijioke O. (2023). The NK cell checkpoint NKG2A maintains expansion capacity of human NK cells. Sci. Rep..

[31] Mariotti F.R., Ingegnere T., Landolina N., Vacca P., Munari E., Moretta L. (2023). Analysis of the mechanisms regulating soluble PD-1 production and function in human NK cells. Front. Immunol..

[32] Hu Y., Hu Q., Li Y., Lu L., Xiang Z., Yin Z., Kabelitz D., Wu Y. (2023). γδ T cells: Origin and fate, subsets, diseases and immunotherapy. Signal Transduct. Target. Ther..

[33] Sivori S., Della Chiesa M., Carlomagno S., Quatrini L., Munari E., Vacca P., Tumino N., Mariotti F.R., Mingari M.C., Pende D. (2020). Inhibitory receptors and checkpoints in human NK cells, Implications for the immunotherapy of cancer. Front. Immunol..

[34] Senju H., Kumagai A., Nakamura Y., Yamaguchi H., Nakatomi K., Fukami S., Shiraishi K., Harada Y., Nakamura M., Okamura H. (2018). Effect of IL-18 on the expansion and phenotype of human natural killer cells: Application to cancer immunotherapy. Int. J. Biol. Sci..

[35] Knuth A., Gabbert H., Dippold W., Klein O., Sachsse W., Bitter-Suermann D., Prellwitz W., Meyer zum Büschenfelde K.H. (1985). Biliary adenocarcinoma. Characterisation of three new human tumor cell lines. J. Hepatol..

[36] Du J., Lv X., Zhang Z., Huang Z., Zhang E. (2023). Revisiting targeted therapy and immunotherapy for advanced cholangiocarcinoma. Front. Immunol..

[37] Kabelitz D., Wesch D., Pitters E., Zöller M. (2004). Potential of human γδ T lymphocytes for immunotherapy of cancer. Int. J. Cancer.

[38] Hayday A.C. (2000). γδ cells: A right time and a right place for a conserved third way of protection. Annu. Rev. Immunol..

[39] Costa G.P., Mensurado S., Silva-Santos B. (2023). Therapeutic avenues for γδ T cells in cancer. J. Immunother. Cancer.

[40] Alnaggar M., Xu Y., Li J., He J., Chen J., Li M., Wu Q., Lin L., Liang Y., Wang X. (2019). Allogenic Vγ9Vδ2 T cell as new potential immunotherapy drug for solid tumor: A case study for cholangiocarcinoma. J. Immunother. Cancer.

[41] Xu Y., Xiang Z., Alnaggar M., Kouakanou L., Li J., He J., Yang J., Hu Y., Chen Y., Lin L. (2021). Allogeneic Vγ9Vδ2 T-cell immunotherapy exhibits promising clinical safety and prolongs the survival of patients with late-stage lung or liver cancer. Cell. Mol. Immunol..

[42] Nakashima M., Tanaka Y., Okamura H., Kato T., Imaizumi Y., Nagai K., Miyazaki Y., Murota H. (2024). Development of Innate-Immune-Cell-Based Immunotherapy for Adult T-Cell Leukemia-Lymphoma. Cells.

[43] Umeyama Y., Taniguchi H., Gyotoku H., Senju H., Tomono H., Takemoto S., Yamaguchi H., Tagod M.S.O., Iwasaki M., Tanaka Y. (2023). Three distinct mechanisms underlying human γδ T cell-mediated cytotoxicity against malignant pleural mesothelioma. Front. Immunol..

[44] Bukowski J.F., Morita C.T., Brenner M.B. (1994). Recognition and destruction of virus-infected cells by human gamma delta CTL. J. Immunol..

[45] Howard J., Zaidi I., Loizon S., Mercereau-Puijalon O., Déchanet-Merville J., Mamani-Matsuda M. (2018). Human Vγ9Vδ2 T Lymphocytes in the Immune Response to. Front. Immunol..

[46] Farrington L.A., Jagannathan P., McIntyre T.I., Vance H.M., Bowen K., Boyle M.J., Nankya F., Wamala S., Auma A., Nalubega M. (2016). Frequent Malaria Drives Progressive Vδ2 T-Cell Loss, Dysfunction, and CD16 Up-regulation During Early Childhood. J. Infect. Dis..

[47] Ho M., Tongtawe P., Kriangkum J., Wimonwattrawatee T., Pattanapanyasat K., Bryant L., Shafiq J., Suntharsamai P., Looareesuwan S., Webster H.K. (1994). Polyclonal expansion of peripheral γδ T cells in human *Plasmodium falciparum* malaria. Infect. Immun..

[48] Jagannathan P., Lutwama F., Boyle M.J., Nankya F., Farrington L.A., McIntyre T.I., Bowen K., Naluwu K., Nalubega M., Musinguzi K. (2017). Vδ2^+^ T cell response to malaria correlates with protection from infection but is attenuated with repeated exposure. Sci. Rep..

[49] Shen L., Huang D., Qaqish A., Frencher J., Yang R., Shen H., Chen Z.W. (2020). Fast-acting γδ T-cell subpopulation and protective immunity against infections. Immunol. Rev..

[50] Rasi V., Wood D.C., Eickhoff C.S., Xia M., Pozzi N., Edwards R.L., Walch M., Bovenschen N., Hoft D.F. (2021). Granzyme A Produced by γ_9_δ_2_ T Cells Activates ER Stress Responses and ATP Production, and Protects Against Intracellular Mycobacterial Replication Independent of Enzymatic Activity. Front. Immunol..

[51] Barnes P.F., Grisso C.L., Abrams J.S., Band H., Rea T.H., Modlin R.L. (1992). γδ T lymphocytes in human tuberculosis. J. Infect. Dis..

[52] Dieli F., Troye-Blomberg M., Ivanyi J., Fournié J.J., Krensky A.M., Bonneville M., Peyrat M.A., Caccamo N., Sireci G., Salerno A. (2001). Granulysin-dependent killing of intracellular and extracellular Mycobacterium tuberculosis by Vγ9/Vδ2 T lymphocytes. J. Infect. Dis..

[53] Cui J., Wang N., Zhao H., Jin H., Wang G., Niu C., Terunuma H., He H., Li W. (2014). Combination of radiofrequency ablation and sequential cellular immunotherapy improves progression-free survival for patients with hepatocellular carcinoma. Int. J. Cancer.

[54] Hwang S., Han J., Baek J.S., Tak E., Song G.W., Lee S.G., Jung D.H., Park G.C., Ahn C.S., Kim N. (2019). Cytotoxicity of human hepatic intrasinusoidal CD56^bright^ natural killer cells against hepatocellular carcinoma cells. Int. J. Mol. Sci..

[55] Kang Y., Han M., Kim M., Hwang H.J., Ahn B.C., Tak E., Song G.W., Hwang S., Koh K.N., Jung D.H. (2023). Cytotoxicity of human hepatic intrasinusoidal γδ T cells depends on phospho-antigen and NK receptor signaling. Anticancer Res..

[56] Tanaka Y., Murata-Hirai K., Iwasaki M., Matsumoto K., Hayashi K., Kumagai A., Nada M.H., Wang H., Kobayashi H., Kamitakahara H. (2018). Expansion of human γδ T cells for adoptive immunotherapy using a bisphosphonate prodrug. Cancer Sci..

