# Supplementary material for: Analysis of the Effector Functions of Vδ2 γδ T Cells and NK Cells against Cholangiocarcinoma Cells
Source: Cells. 2024 Aug 8;13(16):1322. doi: 10.3390/cells13161322 (PMC11352430; doi:10.3390/cells13161322)
Supplement: Supplementary file 1 [file cells-13-01322-s001.zip › cells-3139757-supplementary.pdf]

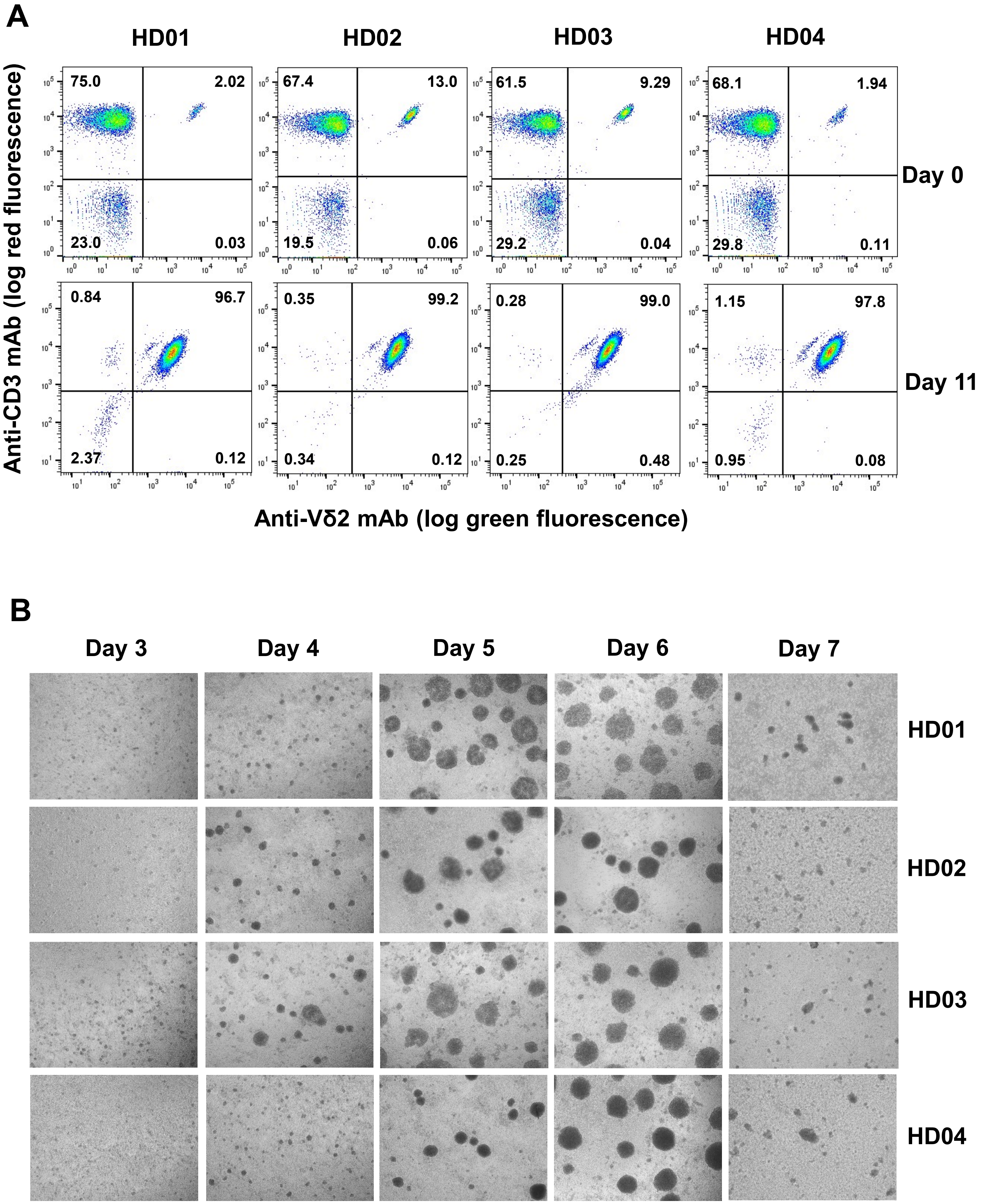
A
HD01
HD02
HD03
HD04
Anti-CD3 mAb (log red fluorescence)
Anti-Vδ2 mAb (log green fluorescence)
Day 0
Day 11
75.0
2.02
23.0
0.03
67.4
13.0
19.5
0.06
61.5
9.29
29.2
0.04
68.1
1.94
29.8
0.11
0.84
96.7
2.37
0.12
0.35
99.2
0.34
0.12
0.28
99.0
0.25
0.48
1.15
97.8
0.95
0.08
B
Day 3
Day 4
Day 5
Day 6
Day 7
HD01
HD02
HD03
HD04

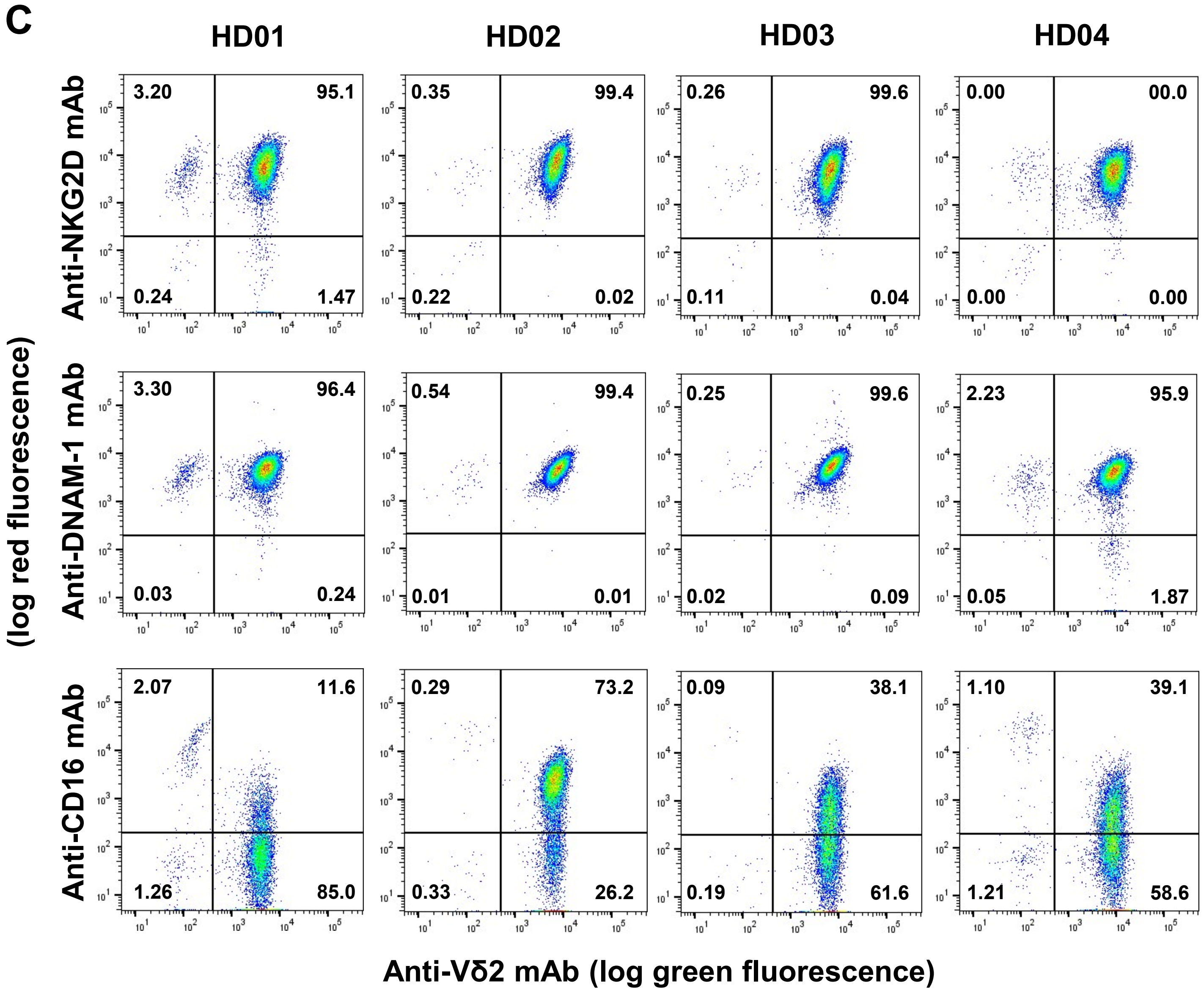
C
HD01
HD02
HD03
HD04
Anti-NKG2D mAb
Anti-DNAM-1 mAb
Anti-CD16 mAb
(log red fluorescence)
3.20
95.1
0.24
1.47
0.35
99.4
0.22
0.02
0.26
99.6
0.11
0.04
0.00
00.0
0.00
0.00
3.30
96.4
0.03
0.24
0.54
99.4
0.01
0.01
0.25
99.6
0.02
0.09
2.23
95.9
0.05
1.87
2.07
11.6
1.26
85.0
0.29
73.2
0.33
26.2
0.09
38.1
0.19
61.6
1.10
39.1
1.21
58.6
Anti-Vδ2 mAb (log green fluorescence)

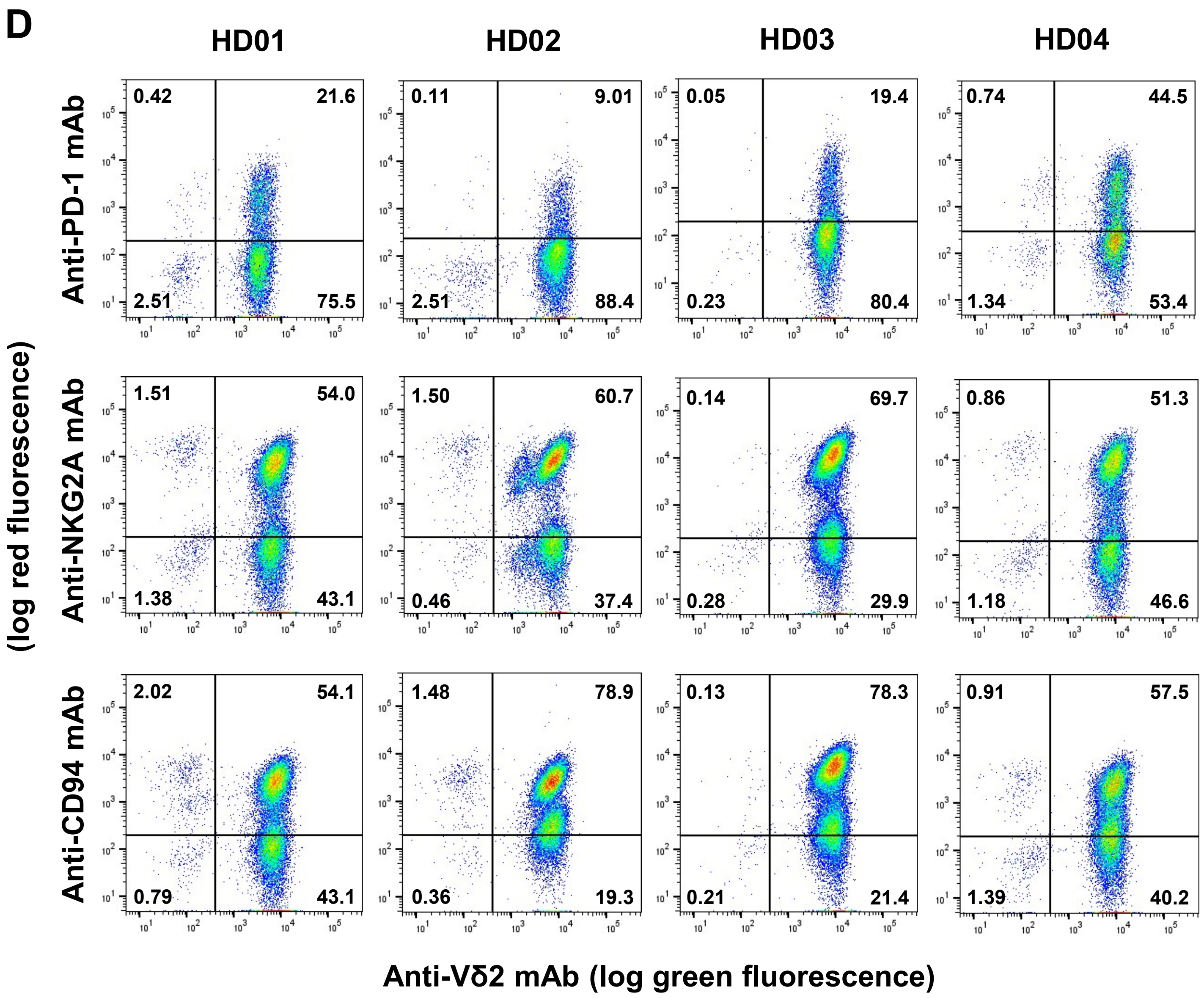

Supplementary Figure S1. Expansion of Vδ2 γδ T cells by PTA/IL-2. (A) Flow cytometric analyses of PTA/IL-2-mediated expansion of Vδ2 γδ T cells derived from healthy donors. PBMC from four healthy donors (HD01-HD04) were stimulated with PTA/IL-2 for 11 days. The cells were analyzed for the expression of CD3 and TCR-Vδ2 before and after expansion using flow cytometry. (B) PTA-mediated clustering of γδ T cells. After stimulation with PTA/IL-2, cell clustering was observed under a microscope equipped with a CCD camera. (C) Flow cytometric analysis of effector molecules on PTA-expanded γδ T cells. Following PTA/IL-2-mediated expansion, the cells were analyzed for the expression of TCR-Vδ2, NKG2D, DNAM-1, and CD16 using flow cytometry. (D) Flow cytometric analysis of co-inhibitory molecules on PTA-expanded γδ T cells. Following PTA/IL-2-mediated expansion, the cells were analyzed for the expression of TCR-Vδ2, PD-1, NKG2A, and CD94 using flow cytometry.

Materials and Methods for Supplementary Figure S1.

(1) Expansion of Vδ2 γδ T cells with PTA/IL-2. Heparinized peripheral blood (10 mL) was placed in a 50 mL polypropylene conical tube (Corning Inc., Corning, NY, USA) containing 10 mL of Dulbecco's phosphate buffered saline (PBS, Shimadzu Diagnostics Corp., Taito-ku, Tokyo, Japan). The diluted blood was layered on 20 mL of Ficoll Paque® Plus (Cytiva, Marlborough, MA, USA) in a 50 mL conical tube. After centrifugation at 600 × g and 37ºC for 30 min in a refrigerated centrifuge (AX-521, TOMY Digital Biology Co., Ltd., Nerima-ku, Tokyo, Japan), the buffy coat was transferred into a 50 mL conical tube containing 35 mL of PBS, followed by centrifugation at 900 × g and 4ºC for 10 min. After removing the supernatant, the cell pellet was dispersed by tapping and resuspended in 13 mL of PBS in a 15 mL conical tube (AGC Techno Glass Co., Ltd., Haibara-gun, Shizuoka, Japan). The conical tube was then centrifuged at 600 × g and 4ºC for 5 min, and the cell pellet was dispersed by tapping and resuspended in Yssel's medium (Kohjin Bio Co., Ltd., Sakado, Saitama, Japan) supplemented with 10% heat-inactivated human AB serum (Kohjin Bio Co., Ltd.) to achieve a cell concentration of 2×10^6 cells/mL. Tetrakis-pivaloyloxymethyl 2-(thiazole-2-ylamino) ethylidene-1,1-bisphosphonate (PTA, Techno Suzuta Co., Ltd., Heiwa-machi, Nagasaki, Japan) was added to reach a concentration of 1 μM. The cell suspension was then placed in 24-well plate (1.5 mL/well, Corning Inc.) and incubated at 37ºC with 5% CO_2 under humidified conditions. After 24 h, interleukin-2 (IL-2, Kyowa Pharmaceutical Industries Co., Ltd., Kita-ku, Osaka, Japan) was added to the cell suspension at a concentration of 10^2 IU/mL. On day 2, the medium was replaced with fresh medium containing 10^2 IU/mL of IL-2. On days 3 and 4, 15 μL of 10^4 IU/mL of IL-2 was added to the cell suspension to achieve a concentration of 100 IU/mL. On days 5 through 9, the cell suspension was split into two portions and supplemented with 10^2 IU/mL of IL-2. On day 11, the cell suspension was transferred into 50 mL conical tubes, which were centrifuged at 600 × g and 4ºC for 5 min. After aspirating the culture supernatant, the cell pellets were dispersed with tapping and resuspended in cryopreservation medium (Takara Bio Inc., Kusatsu, Shiga, Japan) at a cell concentration of 5×10^7 cells/mL. The cell suspension was then transferred into cryopreservation vials (Thermo Fisher Scientific Inc., Waltham, MA, USA), which were placed at -80ºC overnight and subsequently in the liquid nitrogen tank until used as previously described (Tanaka Y.; et al., Expansion of human γδ T cells for adoptive immunotherapy using a bisphosphonate prodrug. *Cancer Sci.* **2018**, *109*: 587-599. https://doi.org/10.1111/cas.13491).

(2) Flow cytometric analysis. PBMC suspensions before and after expansion with PTA/IL-2 were dispensed into the wells of a round bottom 96-well plate to achieve a cell concentration of 2×10^5 cells/100 μL. The plate was centrifuged at 600 × g and 4ºC for 2 min. After removing the supernatants by flipping, the cell pellets were dispersed by vortexing and resuspended in 50 μL of phosphate-buffered saline (PBS)/2% fetal calf serum (FCS) containing 3 μL of fluorescein isothiocyanate (FITC)-conjugated anti-T cell receptor (TCR) Vδ2 mAb (Beckman Coulter Inc., Pasadena, CA, USA) and phycoerythrin (PE)-conjugated anti-cluster of differentiation 3 (CD3) mAb (Thermo Fisher Scientific Inc.), NKG2D, DNAM-1, CD16, NKG2A, or CD94 mAb (BioLegend Japan, Bunkyo-ku, Tokyo, Japan), or PD-1 mAb (MBL Co., Ltd., Minato-ku, Tokyo, Japan), followed by RPE-conjugated anti-mouse Ig Ab (FUJIFILM Wako Pure Chem, Corp., Chuo-ku, Osaka, Japan). After incubating on ice for 15 min, 200 μL of PBS/2% FCS was added to the wells. The plate was then centrifuged at 600 × g and 4ºC for 2 min and the supernatants were removed. After vortexing the plate, 200 μL of PBS/2% FCS was added to the wells. This process was repeated two more times, and the cells were finally resuspended in 200 μL of PBS/2% FCS and analyzed using a FACS Lyrics flow cytometer (Becton, Dickinson and Company, Franklin Lakes, NJ, USA).

(3) Microscopic analysis. PBMC before and after expansion with PTA/IL-2 were observed under a microscope equipped with a 4 × objective lens and a 10 × eyepiece lens. The images were captured using cellSens softoware ver. 2.3 (Olympus Corp., Hachioji, Tokyo, Japan).

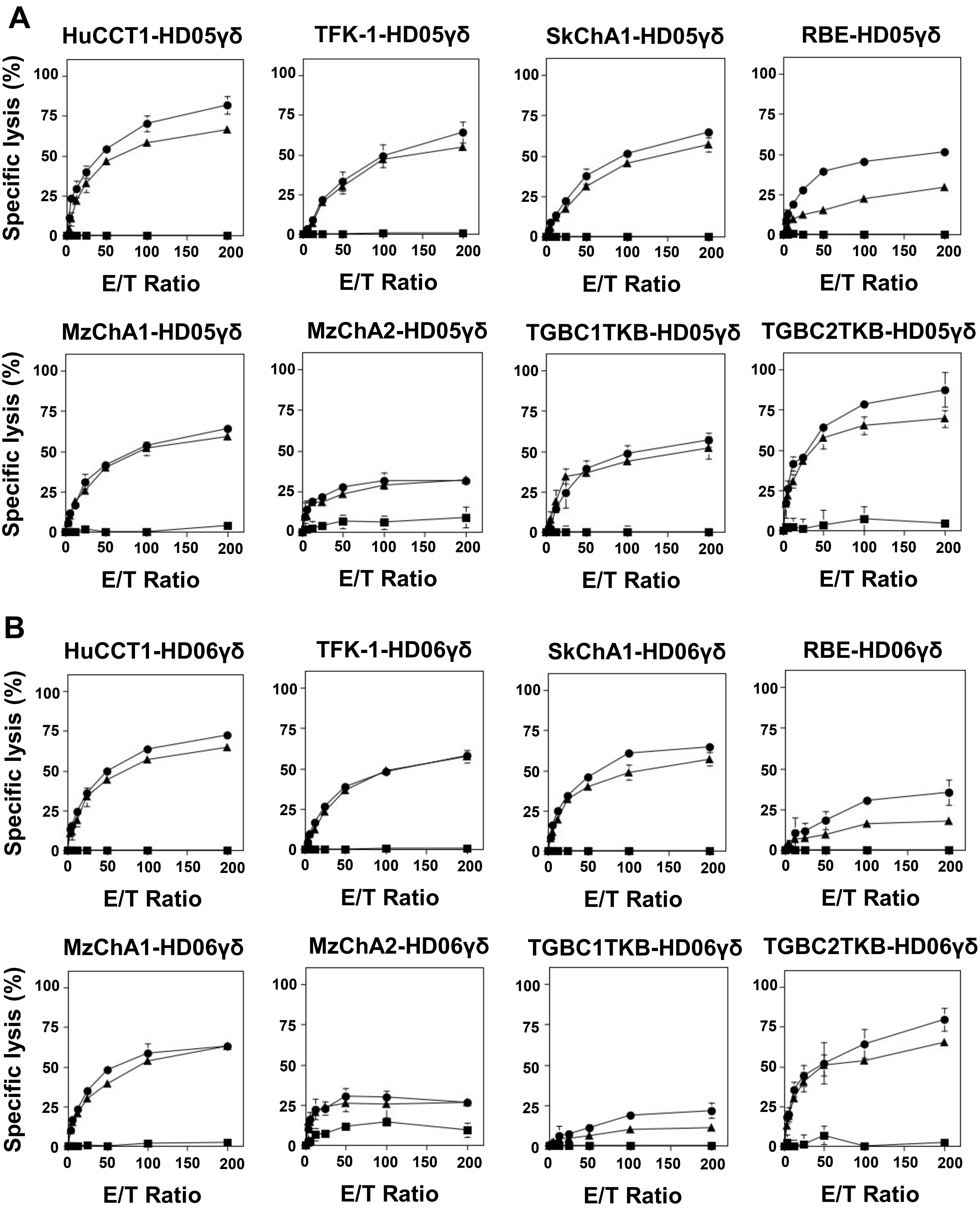

Supplementary Figure S2. Cytotoxicity displayed by Vδ2 γδ T cells against CCA cell lines pretreated with 0 (■), 100 (▲), or 500 nM (●) of PTA. (A) γδ cells derived from a healthy donor HD05. (B) γδ cells derived from a healthy donor HD06.

C

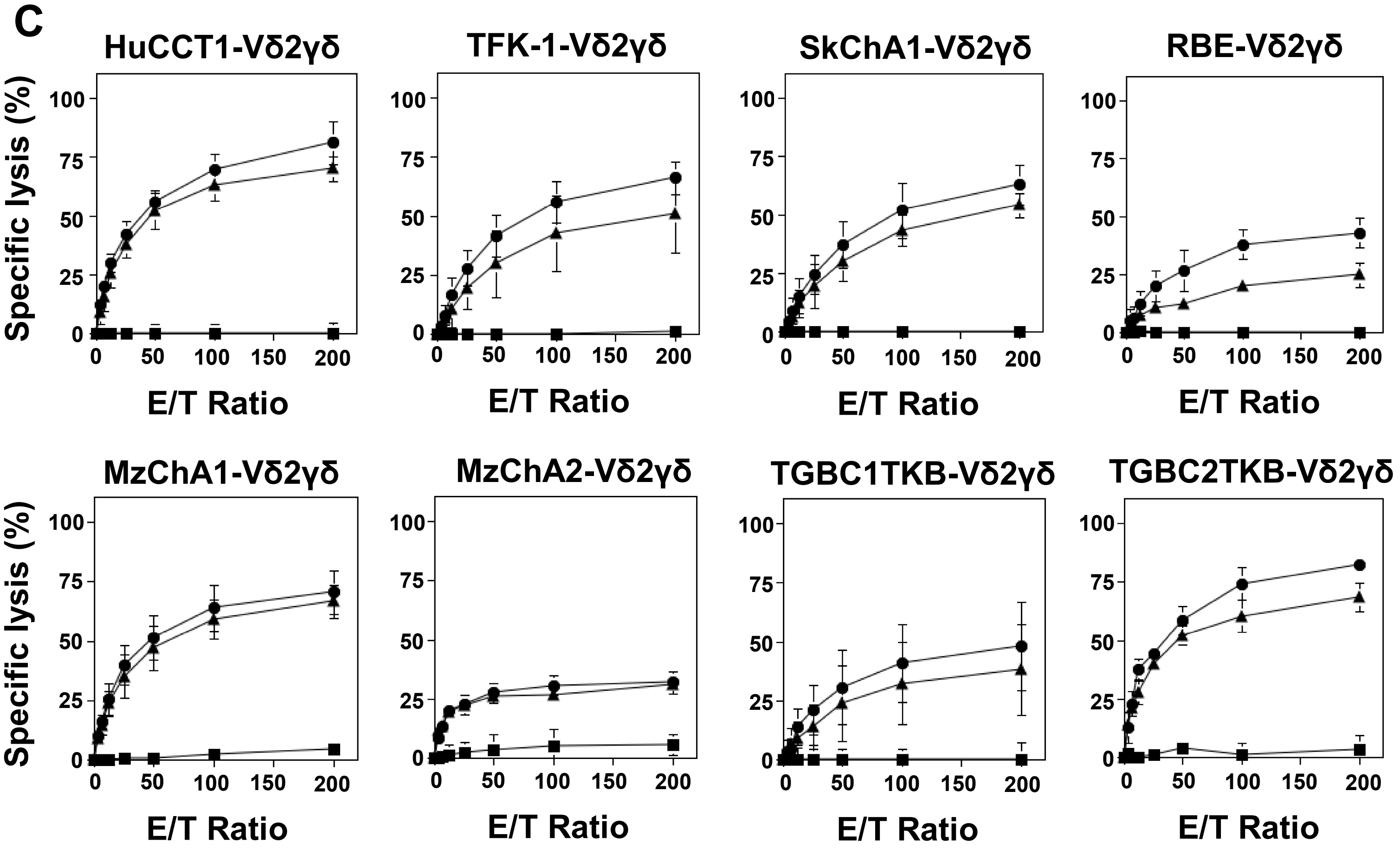

Supplementary Figure S2. Cytotoxicity displayed by Vδ2 γδ T cells against CCA cell lines pretreated with 0 (■), 100 (▲), or 500 nM (●) of PTA. (C) Average cellular cytotoxicity against CCA cell lines exhibited by Vδ2 γδ cells derived from healthy donors HD01, HD02, HD05, and HD06. The specific lysis percentages of HD01, HD02, HD05, and HD06 were combined and depicted as line graphs.

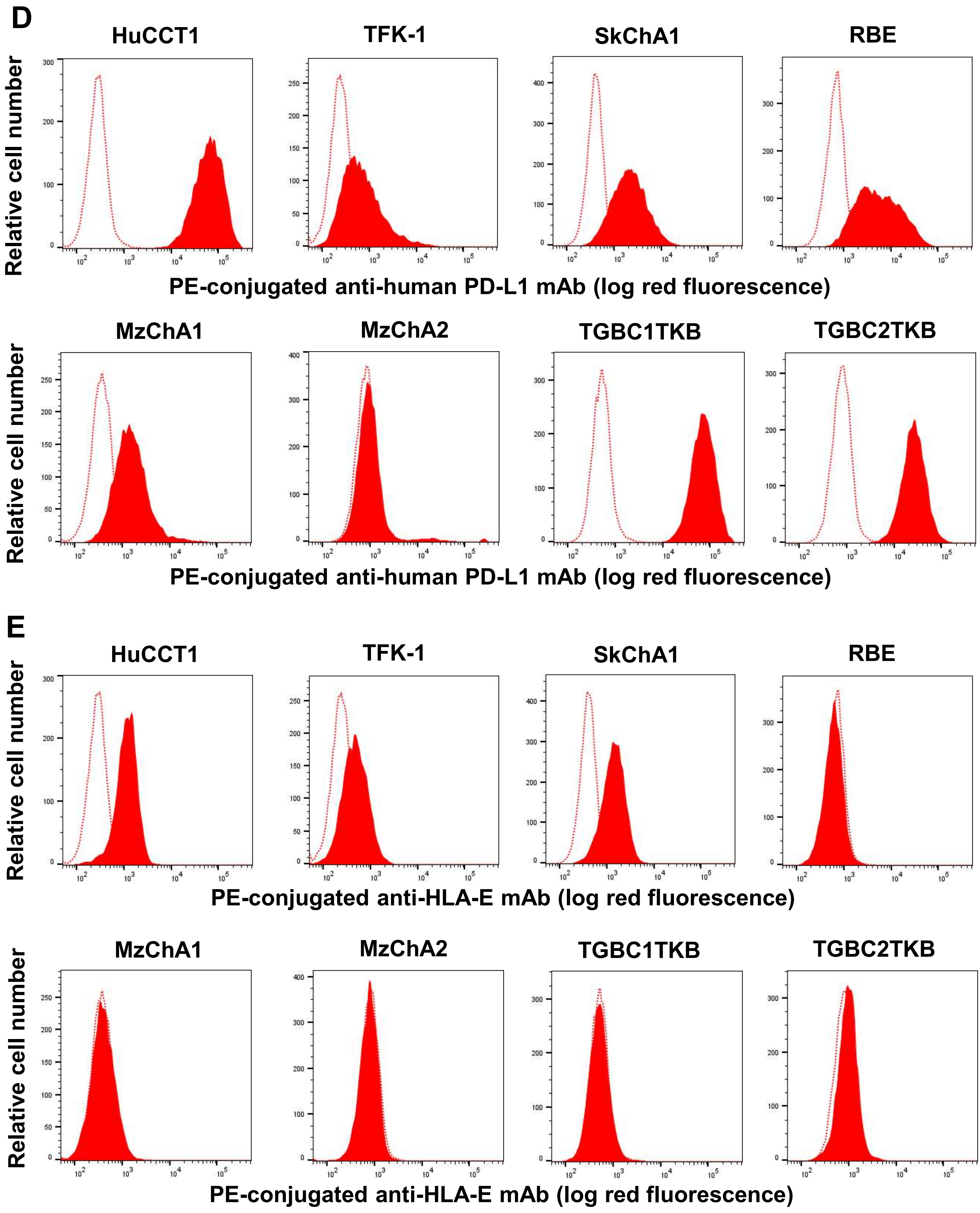

Supplementary Figure S2. Cytotoxicity displayed by Vδ2 γδ T cells against CCA cell lines pretreated with 0 (■), 100 (▲), or 500 nM (●) of PTA. Flow cytometric analysis of the expression co-inhibitory molecules on CCA cell lines. (D) PD-L1. (E) HLA-E.

Materials and Methods for Supplementary Figure S2.

(1) Maintenance of human CCA cell lines

Eight human CCA cell lines were utilized in this study. HuCCT1 was obtained from the Japanese Collection of Research Bioresources (JCRB) Cell Bank, National Institutes of Biomedical Innovation, Health and Nutrition (Ibaraki, Osaka, Japan), It was originally established from malignant ascites of a patient with moderately differentiated adenocarcinoma derived from the intrahepatic bile duct tree. TFK-1, RBE, TGBC1TKB, and TGBC2TKB were acquired from the RIKEN BRC Cell Bank (Tsukuba, Ibaraki, Japan). TFK-1 was isolated from extrahepatic bile duct carcinoma and RBE was established from intrahepatic cholangiocarcinoma. TGBC1TKB and TGBC2TKB were isolated from a patient with gallbladder carcinoma metastasized to lymph nodes. Mz-ChA-1, Mz-ChA-2, and Sk-ChA-1 were obtained from Dr. Hidenori Tanaka (Kyoto University, Kyoto, Japan) and originally from Dr. Alexander Knuth (Krankenhaus Nordwest, Frankfurt, Germany). Mz-ChA-1 and Mz-ChA-2 were initially isolated from gallbladder adenocarcinoma metastases, while SK-ChA-1 was established from malignant ascites of a patient with primary adenocarcinoma of the extrahepatic biliary tree[1]. HuCCT1 and TFK-1 were cultured in RPMI1640 medium supplemented with 10% FCS (Merck KGaA), 10^{-5} M 2-mercaptoethanol, 100 U/mL of penicillin (Meiji Seika Pharma Co., Ltd., Chuo-ku, Tokyo, Japan), and 100 μg/mL streptomycin (Meiji Seika Pharma Co., Ltd.). The remaining cell lines were cultured in DMEM medium (Merck KGaA) supplemented with 10% FCS (Merck KGaA), 100 U/mL of penicillin, and 100 μg/mL streptomycin. Cell cultures were maintained in a logarithmic phase at 37°C in a humidified atmosphere containing 5% CO_2.

1. Knuth A, Gabbert H, Dippold W, Klein O, Sachsse W, Bitter-Suermann D, Prellwitz W, Meyer zum Büschenfelde KH. Biliary adenocarcinoma. Characterisation of three new human tumor cell lines. J Hepatol. 1985;1(6):579-96. doi: 10.1016/s0168-8278(85)80002-7. PMID: 4056357.

(2) Time-resolved fluorescence-based short-term cellular cytotoxicity assay

Short-term γδ T cell-mediated cellular cytotoxicity was assessed using a non-radioactive cellular cytotoxicity assay kit (Techno Suzuta Co., Ltd.). Human CCA cells (1×10^6 cells/mL) in 15 mL conical tubes were treated with 0 nM, 100 nM, or 500 nM of PTA at 37°C with 5% CO_2 for 2 h and then pulsed with 25 μM bis(butyryloxymethyl) 4’-(hydroxymethyl)-2,2’:6’,2”-terpyridine-6,6”-dicarboxylate (BM-HT, Techno Suzuta Co., Ltd.) at 37°C with 5% CO_2 for 15 min. During incubation, BM-HT was hydrolyzed by intracellular esterases to yield 4’-(hydroxymethyl)-2,2’:6’,2”-terpyridine-6,6”-dicarboxylate (HT) (40). To the conical tubes, 5 mL of complete RPMI140 medium was added, and the tubes were centrifuged at $600\times g$ at 4°C for 5 min. After removing the supernatants, the cell pellets were dispersed by tapping and resuspended in 5 mL of complete RPMI1640 medium. The cells were washed two more times and resuspended in 5 mL of complete RPMI1640 medium, from which 2 mL were transferred into a new 15 mL conical tube containing 6 mL of complete RPMI1640 medium. Tumor cell suspensions (5×10^3 cells/100 μL) were dispensed into wells of a 96-well round-bottom plate, to which 100 μL each of Vδ2 γδ T cells were added at E/T ratios of 0:1, 3.125:1, 6.25:1, 12.5:1, 25:1, 50:1, 100:1, and 200:1. The plate was centrifuged at $200\times g$ at ambient temperature for 2 min and then incubated at 37°C with 5% CO_2 for 40 min. Detergent (Techno Suzuta Co., Ltd.) was added to the wells to achieve a final concentration of 5×10^{-5} M for the measurement of the maximum release. After mixing the cell suspensions, the plate was centrifuged at $600\times g$ for 2 min, and the supernatants (25 μL each) were transferred to a new 96-well round-bottom plate containing 250 μL of europium (Eu) solution (Techno Suzuta Co., Ltd.). After mixing the Eu/HT complex solution, 200 μL samples were transferred to a 96-well optical plate (Thermo Fisher Scientific Inc.). Time-resolved fluorescence was measured using a NIVO multi-plate reader (Revvity Inc., Yokohama, Kanagawa, Japan). All measurements were conducted in triplicate. Specific lysis (%) was calculated as $100\times$[experimental release (counts)–spontaneous release (counts)]/[maximum release (counts)–spontaneous release (counts)].

Materials and Methods for Supplementary Figure S2.

(3) Human cholangiocarcinoma cells were dispensed into the wells of a round bottom 96-well plate to achieve a cell concentration of 2×10^5 cells/100 µL. The plate was centrifuged at $600 \times g$ and 4ºC for 2 min. After removing the supernatants by flipping, the cell pellets were dispersed by vortexing and resuspended in 50 µL of phosphate-buffered saline (PBS)/2% fetal calf serum (FCS) containing 3 µL of anti-human PD-L1 mAb (27A2, MBL, Minato-ku, Tokyo, Japan), followed by RPE-conjugated anti-mouse Ig Ab (FUJIFILM Wako Pure Chem, Corp., Chuo-ku, Osaka, Japan), or 3 µL of phycoerythrin (PE)-conjugated anti-NKG2A mAb (BioLegend Japan, Bunkyo-ku, Tokyo, Japan). After incubating on ice for 15 min, 200 µL of PBS/2% FCS was added to the wells. The plate was then centrifuged at $600 \times g$ and 4ºC for 2 min and the supernatants were removed. After vortexing the plate, 200 µL of PBS/2% FCS was added to the wells. This process was repeated two more times, and the cells were finally resuspended in 200 µL of PBS/2% FCS and analyzed using a FACS Lyrics flow cytometer (Becton, Dickinson and Company, Franklin Lakes, NJ, USA).

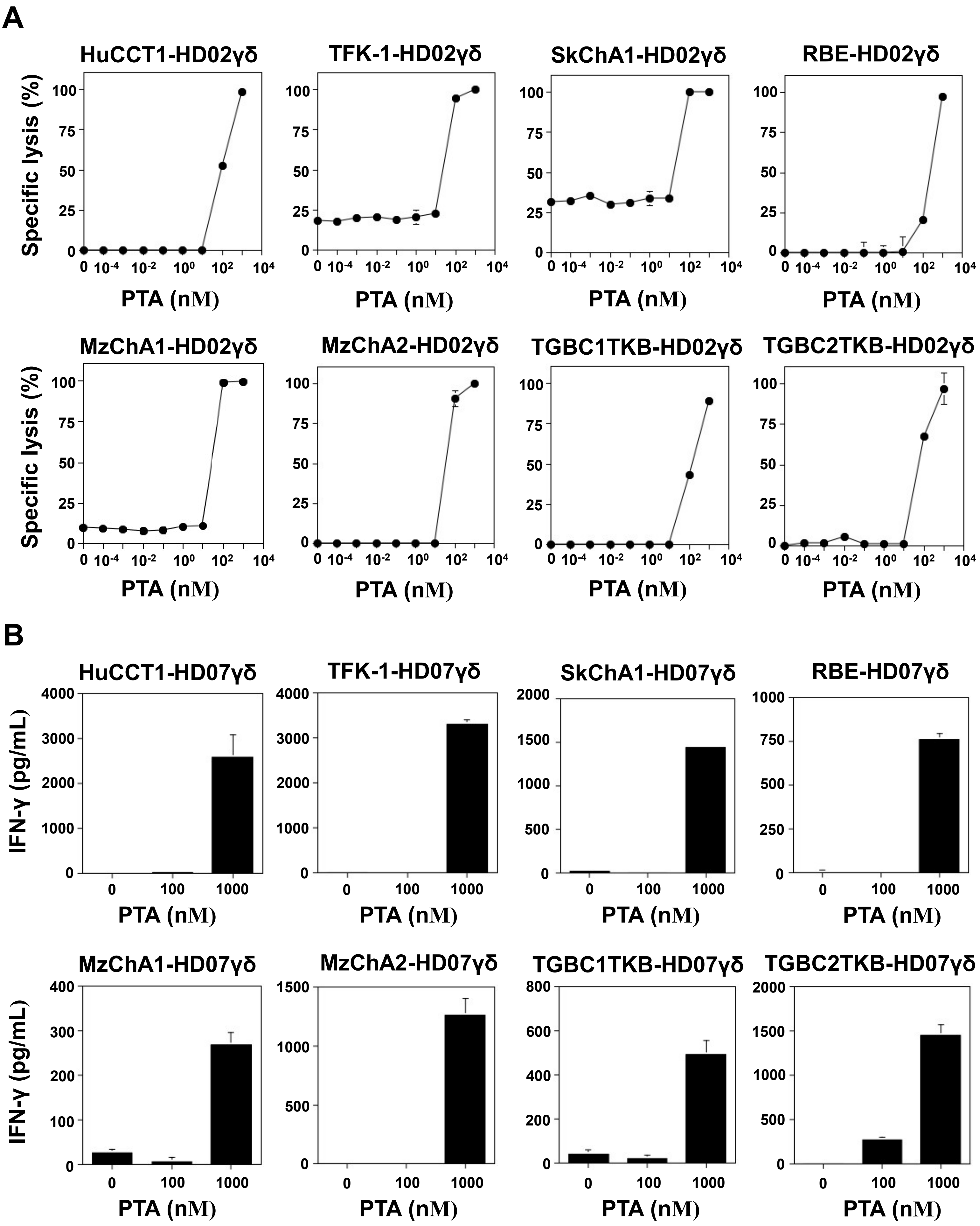

Supplementary Figure S3. Effect of PTA on the effector functions of Vδ2 γδ T cells. (A) Effect of PTA on the long-term cellular cytotoxicity of Vδ2 γδ T cells. (B) Effect of PTA on the secretion of IFN-γ from Vδ2 γδ T cells against CCA cell lines.

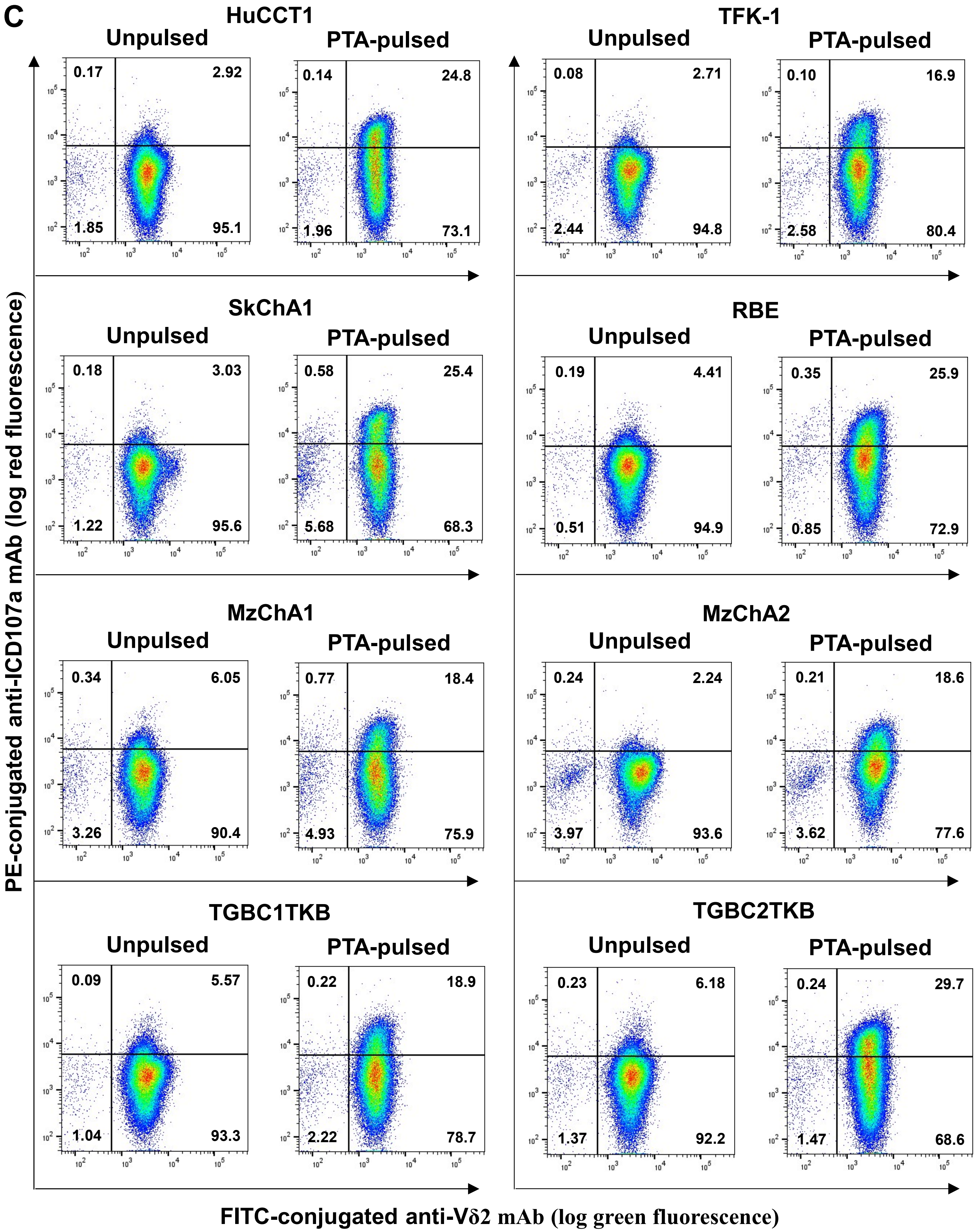

Supplementary Figure S3. Effect of PTA on the effector functions of Vδ2 γδ T cells. (C) Effect of PTA on the degranulation of HD07 Vδ2 γδ T cells when incubated with cholangiocarcinoma cells pretreated with 0 μM (Unpulsed) or 1 μM PTA (PTA-Pulsed).

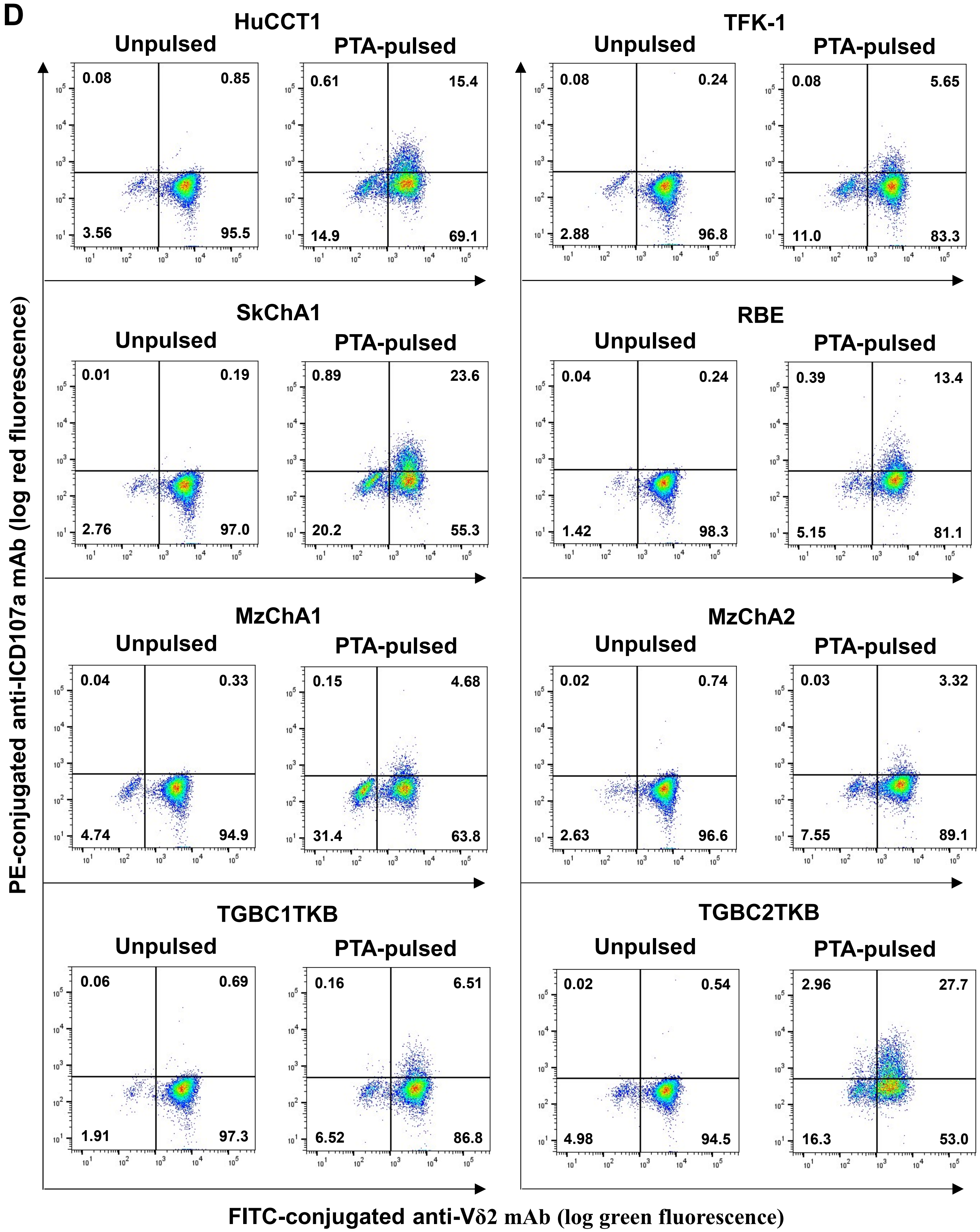

Supplementary Figure S3. Effect of PTA on the effector functions of Vδ2 γδ T cells. (D) Effect of PTA on the IFN-γ secretion of HD07 Vδ2 γδ T cells when incubated with cholangiocarcinoma cells pretreated with 0 μM (Unpulsed) or 1 μM PTA (PTA-pulsed).

Materials and Methods for Supplementary Figure S3.

(1) Vδ2 γδ T cells mediated long-term cytotoxicity assay to assess the effect of PTA. CCA cells were dispensed into the wells of a 96-well plate (2×10^4 cells/200 µL/well) and incubated at 37ºC with 5% CO_2 overnight. After aspirating the culture supernatants, CCA cells were pulsed with 100 µL of 0, 0.1, 1, 10, and 100 pM, and 1, 10, and 100 nM, and 1 µM of PTA at 37ºC with 5% CO_2 for 2 h. Subsequently, the supernatants were aspirated, and 8×10^5 cells/100 µL of Vδ2 γδ T cells were added to the wells at an E/T ratio of 40:1. The plate was then incubated at 37ºC with 5% CO_2 for 72 h. Afterward, the wells were washed three times with 200 µL of RPMI1640 medium supplemented with 10% heat-inactivated FCS. Next, 100 µL of the medium was added to the wells, followed by the addition of 100 µL of CellTiter-Glo Reagent. The wells were mixed well and transferred to a Perkin Elmer OptiPlate 96 plate, and luminescence was measured using a NIVO multi-plate reader (Revvity Inc.).

(2) ELISA for IFN-γ. CCA cells were dispensed into the wells of a 96-well plate (1.6×10^5 cells/200 µL/well), followed by overnight incubation at 37ºC with 5% CO_2. After aspirating the culture supernatants, CCA cells were exposed to 100 µL of PTA at concentrations of 0, 100, and 1,000 nM at 37ºC with 5% CO_2 for 4 h. Subsequently, the supernatants were aspirated, and 1.6×10^5 Vδ2 γδ T cells in 200 µL were added to the wells at an E/T ratio of 1:1. The plate was then incubated at 37ºC with 5% CO_2 overnight. The resulting supernatants were transferred into a new round-bottom 96-well plate, which was then placed at -80ºC overnight. Upon thawing, the supernatants were analyzed for IFN-γ using a human IFN-γ ELISA kit (Thermo Fisher Scientific Inc.) following the manufacturer's protocol. In brief, a capture mAb was diluted with water to achieve a concentration of 1 µg/mL, and 100 µL of this solution was dispensed into wells of an ImmunoPlate (Thermo Fisher Scientific, Inc.). After overnight incubation at room temperature, the plate was washed four times with 300 µL of PBS/0.05% Tween-20. To the wells, 300 µL of PBS/1% BSA was added and left at room temperature for 1 h. After washing the plate four times with 300 µL of PBS/0.05% Tween-20, 100 µL of the supernatants were dispensed into the plate and left at room temperature for 2 h. Following four washes with 300 µL of PBS/0.05% Tween-20, 100 µL of 1 µg/mL biotin-conjugated detection mAb in PBS/0.05% Tween-20/0.1% BSA was dispensed into wells and left at room temperature for 2 h. After washing the plate four times with 300 µL of PBS/0.05% Tween-20, 100 µL of horseradish-conjugated avidin in PBS/0.05% Tween-20/0.1% BSA was dispensed into wells and left at room temperature for 30 min. Following another four washes with 300 µL of PBS/0.05% Tween-20, 100 µL of 2,2'-azino-bis(3-ethylbenzothiazoline-6-sulfonic acid (ABTS) was dispensed into wells. After a 5-minute incubation, the absorbance at 405 nm was measured using a NIVO multi-plate reader (Revvity Inc.).

(3) Degranulation assay. CCA cells (1 x 10^6 cells/mL) were pretreated with either 0 or 1 µM PTA at 37ºC with 5% CO_2 for 2 h and washed with 200 µL of complete RPMI1640 medium. The cells were resuspended in complete RPMI1640 medium to give a cell concentration of 1 x 10^7 cells/mL. The cell suspensions were dispensed into the wells of a 96-well round-bottom plate (2 x 10^5 cells/20 µL), to which were added 2 x 10^5 HD07 Vδ2 γδ T cells in 20 µL of the complete RPMI1640 medium. To the wells were added 10 µL of PE-conjugated with anti-CD107a mAb (BioLegend Japan, Bunkyo-ku, Tokyo, Japan) and the plate was incubated at 37ºC with 5% CO_2 for 2 h. The cells were washed three times and resuspended in 47 µL of PBS/2% FCS, to which was added 3 µL of FITC-conjugated anti-Vδ2 mAb (Beckman Coulter Inc., Pasadena, CA). After the plate was placed on ice for 15 min, the cells were washed three times and resuspended in 200 µL of PBS/2% FCS and were analyzed for the expression of CD107a and Vδ2 using a FACS Lyrics flow cytometer (Becton, Dickinson and Company, Franklin Lakes, NJ, USA).

Materials and Methods for Supplementary Figure S3.

(4) Intracellular staining of IFN-γ. CCA cells (1 x 10^6 cells/mL) were pretreated with either 0 or 1 μM PTA at 37°C with 5% CO_2 for 2 h and washed with 200 μL of complete RPMI1640 medium. The cells were resuspended in complete RPMI1640 medium to give a cell concentration of 1 x 10^7 cells/mL. The cell suspensions were dispensed into the wells of a 96-well round-bottom plate (2 x 10^5 cells/20 μL), to which were added 2 x 10^5 HD07 Vδ2 γδ T cells in 20 μL of the complete RPMI1640 medium. To the wells were added 10 μL of PE-conjugated with anti-CD107a mAb (BioLegend Japan, Bunkyo-ku, Tokyo, Japan) and the plate was incubated at 37°C with 5% CO_2 for 2 h. The cells were washed three times and resuspended in 47 μL of PBS/2% FCS, to which was added 3 μL of FITC-conjugated anti-Vδ2 mAb (Beckman Coulter Inc., Pasadena, CA). After the plate was placed on ice for 15 min, the cells were washed three times and resuspended in 200 μL of PBS/2% FCS and were analyzed for the expression of CD107a and Vδ2 using a FACS Lyrics flow cytometer (Becton, Dickinson and Company, Franklin Lakes, NJ, USA).

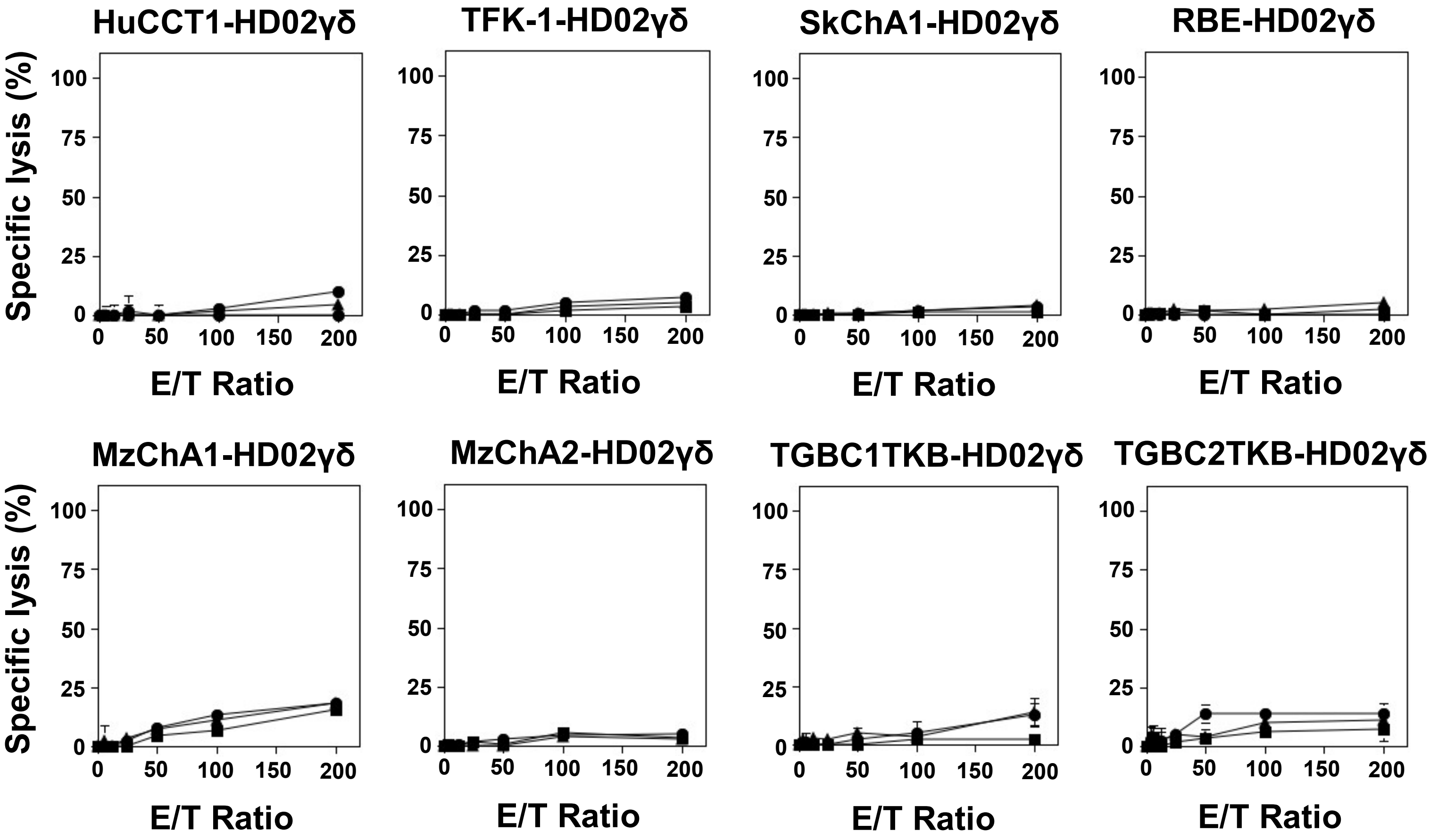

Supplementary Figure S4. Effect of anti-EGFR mAb on CD16-mediated cytotoxicity by Vδ2 γδ T cells against CCA cells in short-term cellular cytotoxicity assay. CCA cells were pretreated with 0 (■), 1 (▲), or 10 µg/mL (●) of anti-EGFR mAb before being challenged by γδ T cells, and cellular cytotoxicity was assessed using a short-term assay system.

Materials and Methods for Supplementary Figure S4.

Short-term ADCC by Vδ2 γδ T cells against CCA cells was assessed using a non-radioactive cellular cytotoxicity assay kit (Techno Suzuta Co., Ltd.) following the manufacturer's protocol, as detailed in the Materials and Methods section of Supplementary Figure S2. To investigate the effect of anti-EGFR mAb on short-term γδ T cell-mediated cellular cytotoxicity, CCA cells were treated with 0, 1, or 10 µg/mL of mAb for 15 min before labeling with BM-HT. A time-resolved fluorescence-based assay was conducted at various effector-to-target (E/T) ratios: 0:1, 3.125:1, 6.25:1, 12.5:1, 25:1, 50:1, 100:1, and 200:1.

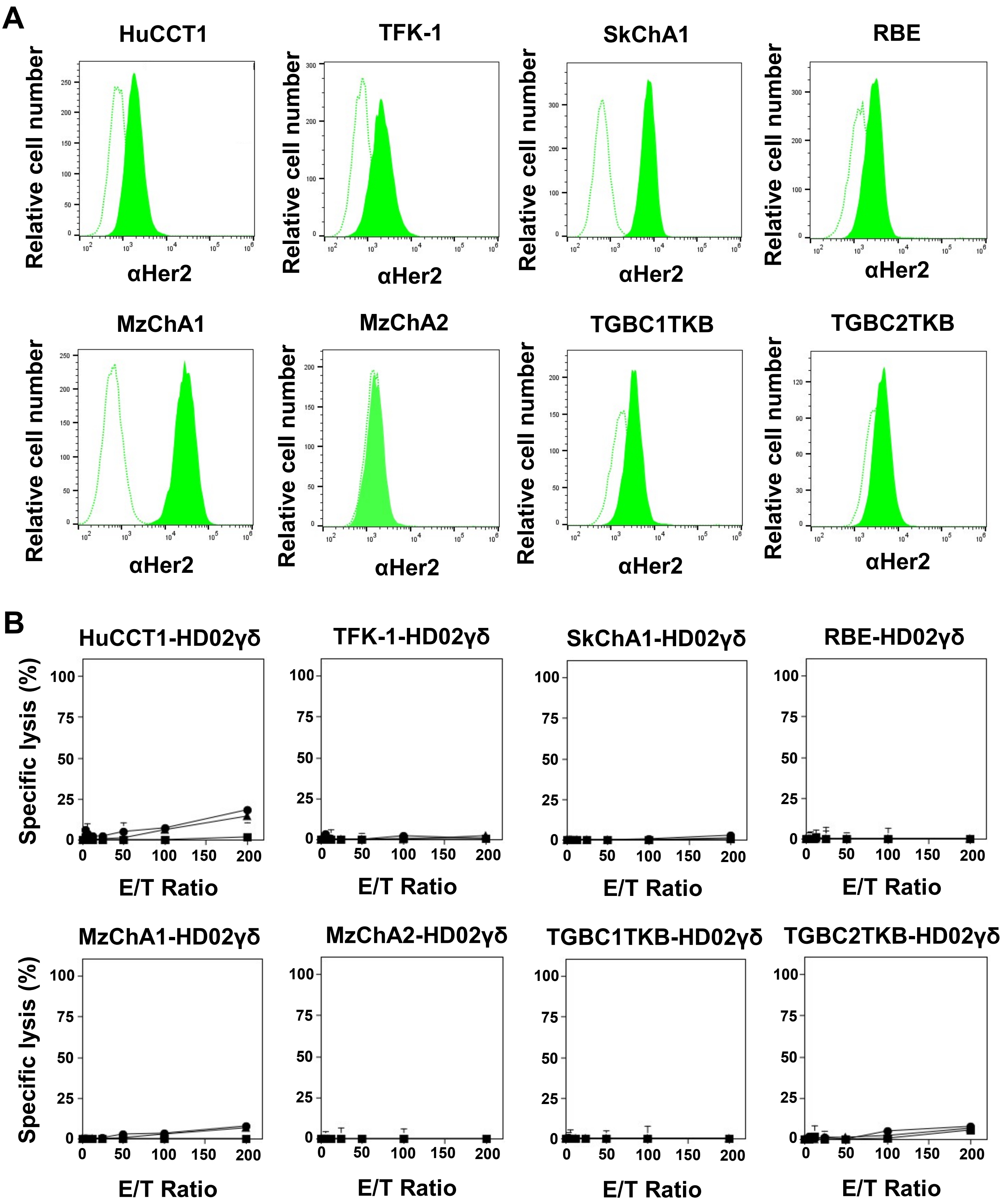

Supplementary Figure S5. CD16-mediated cytotoxicity against CCA cells by Vδ2 γδ T cells. (A) Expression of Her2 on cholangiocarcinoma cells. (B) Effect of 0 (■), 1 (▲), or 10 µg/mL (●) of anti-Her2 mAb on the cytotoxic activity of Vδ2 γδ T cells against cholangiocarcinoma cell lines in a short-time cytotoxicity assay.

Materials and Methods for Supplementary Figure S5.

(1) Flow cytometric analysis

CCA cells were examined for the expression of Her2. The cells were dispensed into wells of a round-bottom 96-well plate at a concentration of 2×10^5 cells/100 µL. The plate was centrifuged at 600 × g and 4ºC for 2 min. After flipping to remove the supernatants, cell pellets were dispersed by vortexing and then resuspended in 50 µL of phosphate-buffered saline (PBS)/2% fetal calf serum (FCS), supplemented with 3 µL of biotin-conjugated anti-Her2 mAb and then GFP-conjugated biotin-binding protein. Following a 15-minute incubation on ice, 200 µL of PBS/2% FCS were added to the wells. The plate was centrifuged again at 600 × g and 4ºC for 2 min, and supernatants were removed. After vortexing the plate, 200 µL of PBS/2% FCS were added to the wells, and this process was repeated two more times. The cells were finally resuspended in 200 µL of PBS/2% FCS and analyzed using a FACS Lyrics flow cytometer (Becton, Dickinson and Company, Franklin Lakes, NJ, USA).

(2) Short-term ADCC by Vδ2 γδ T cells against CCA cells was assessed using a non-radioactive cellular cytotoxicity assay kit (Techno Suzuta Co., Ltd.) following the manufacturer's protocol, as detailed in the Materials and Methods section of Supplementary Figure S2. To investigate the effect of anti-Her2 mAb on short-term γδ T cell-mediated cellular cytotoxicity, CCA cells were treated with 0, 1, or 10 µg/mL of mAb for 15 min before labeling with BM-HT. A time-resolved fluorescence-based assay was conducted at various effector-to-target (E/T) ratios: 0:1, 3.125:1, 6.25:1, 12.5:1, 25:1, 50:1, 100:1, and 200:1.

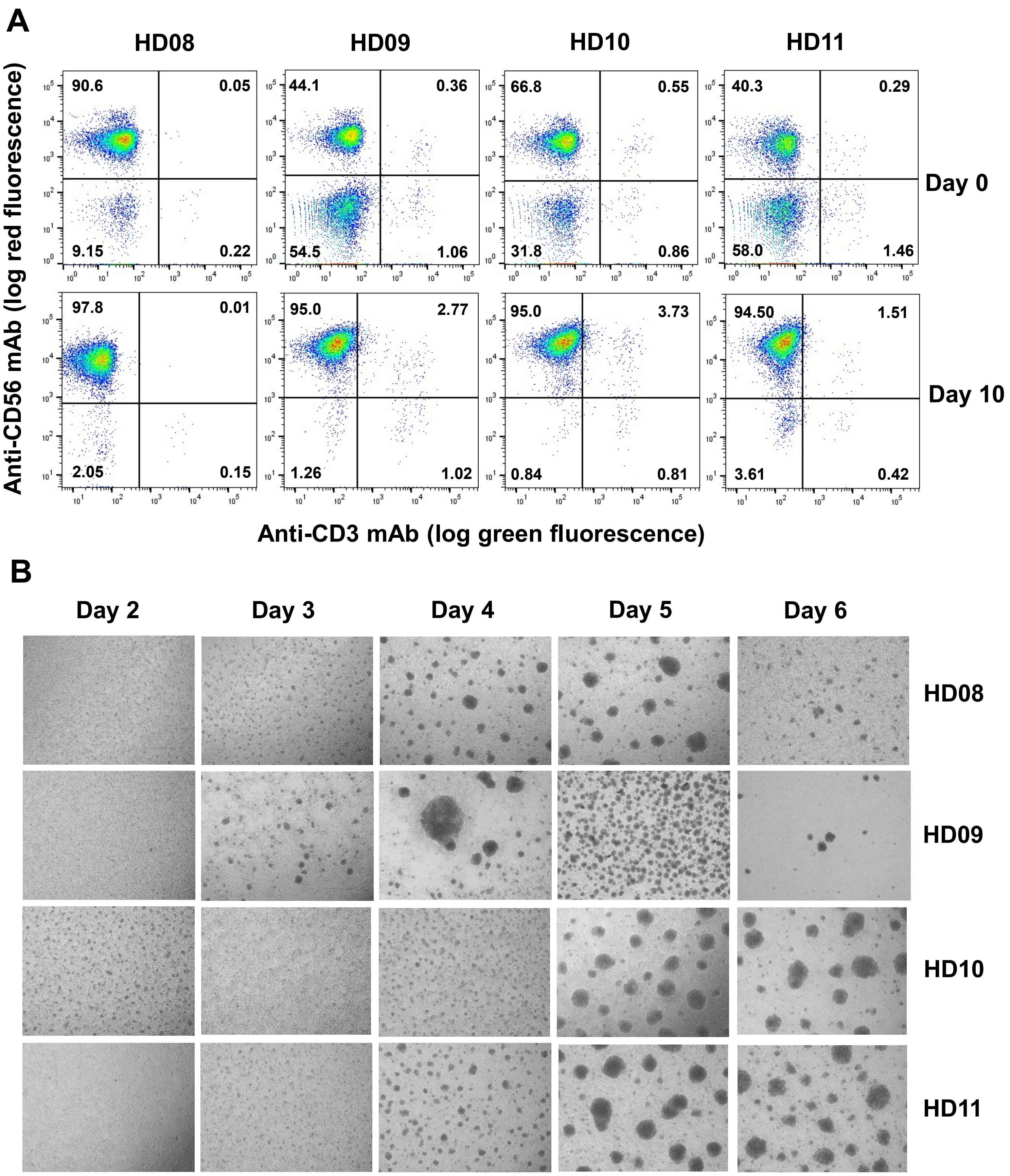

Supplementary Figure S6. Expansion of NK cells by IL-2/IL-18. (A) Flow cytometric analyses illustrating IL-2/IL-18-mediated expansion of NK T cells derived from healthy donors. $CD3^-$ fractions of PBMC after separation with anti-CD3 mAb-conjugated MACS beads were stained with PE-conjugated anti-CD3 mAb and FITC-conjugated anti-CD56 mAb and subjected to flow cytometric analysis. Lymphocyte fractions on forward scattering versus side scattering dot plots were analyzed. (B) IL-2/IL-18-induced clustering of NK cells. $CD3^-$ PBMC stimulated with IL-2/IL-18 were observed under a microscope.

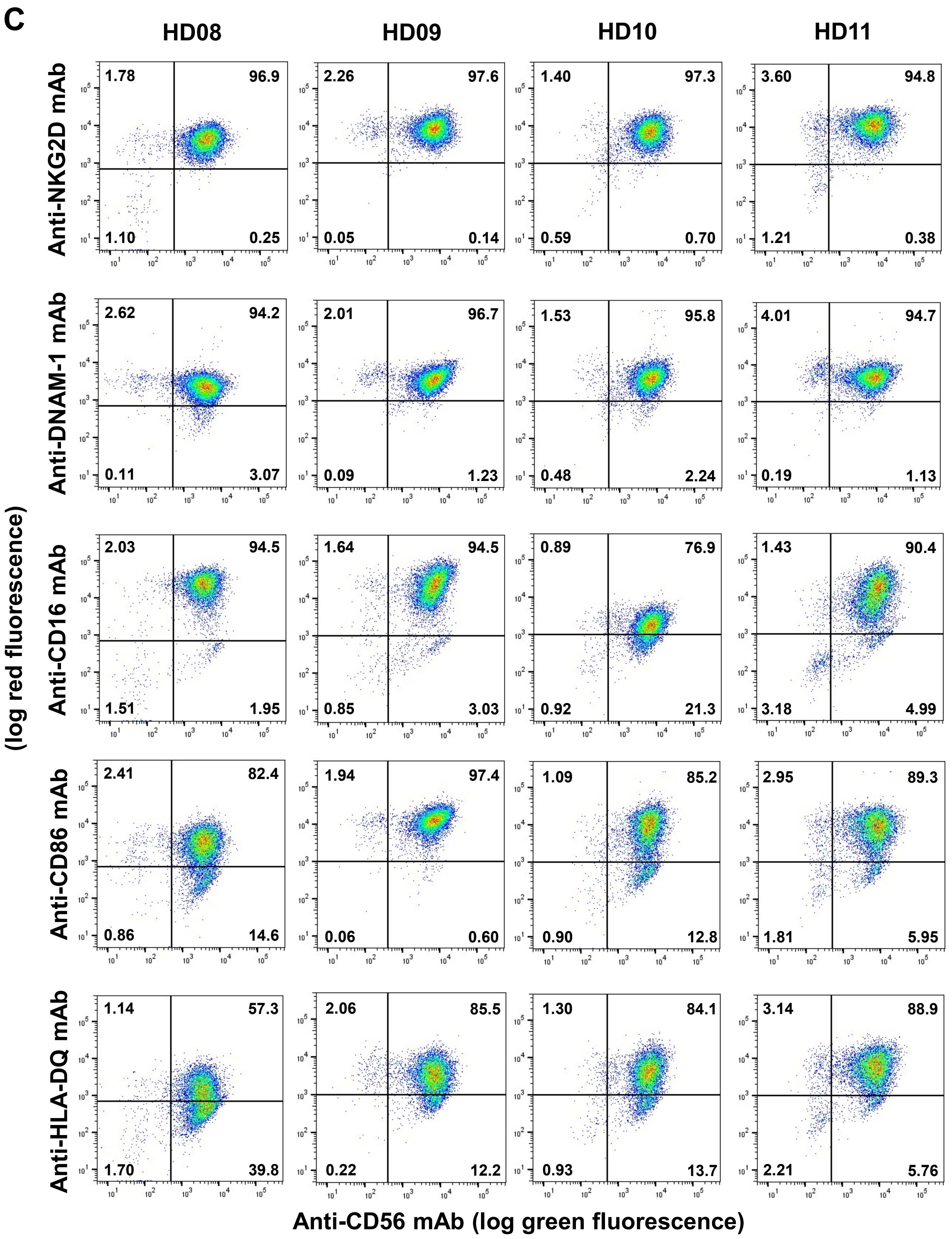

Supplementary Figure S6. Expansion of NK cells by IL-2/IL-18. (C) Flow cytometric analyses of effector molecules on IL-2/IL-18-induced expansion of NK cells derived from healthy donors.

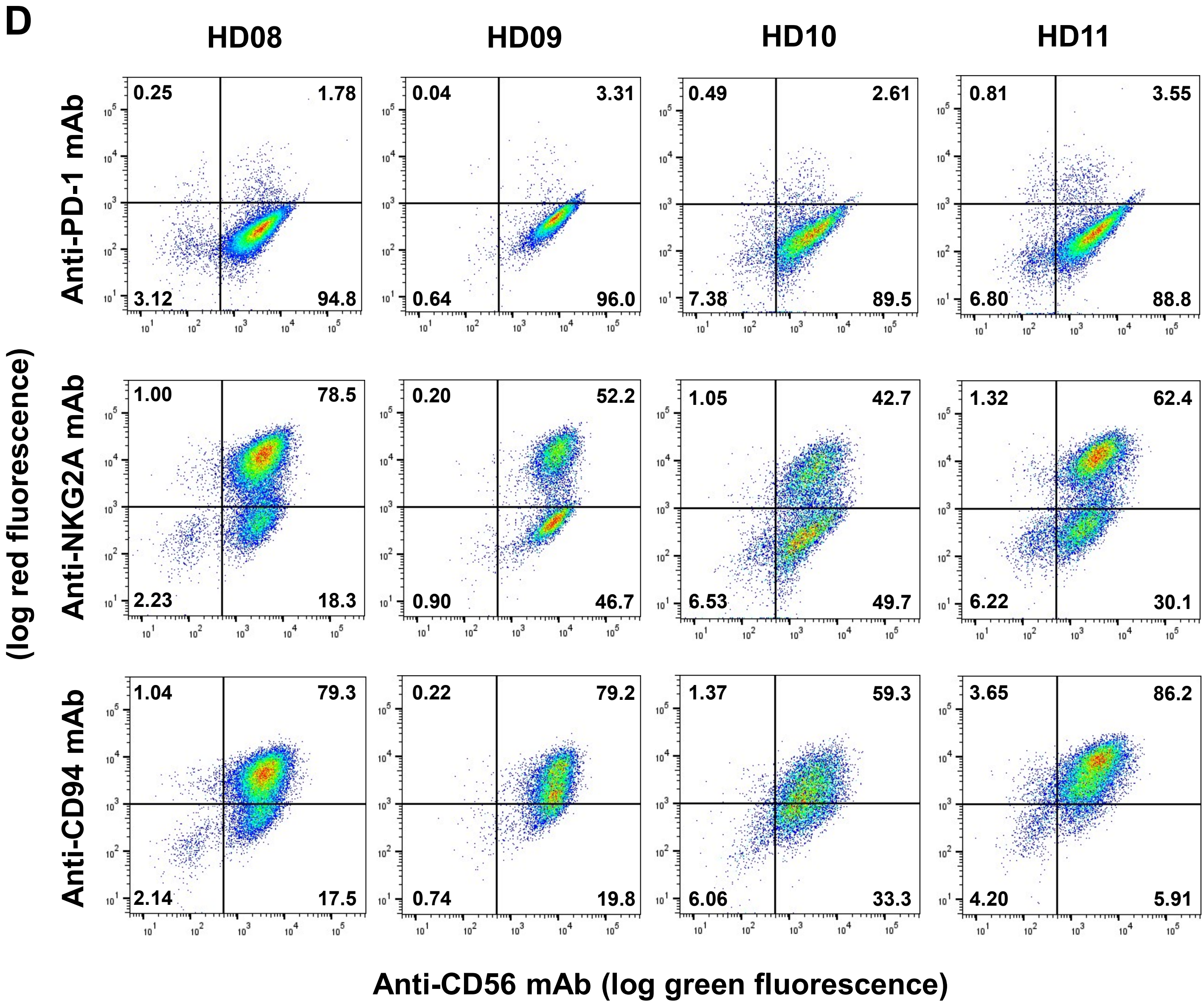

Supplementary Figure S6. Expansion of NK cells by IL-2/IL-18. (D) Flow cytometric analyses of co-inhibitory molecules on IL-2/IL-18-induced expansion of NK cells derived from healthy donors.

Materials and Methods for Supplementary Figure S6.

(1) Expansion of NK cells by IL-2/IL-18. Peripheral blood mononuclear cells (PBMCs) derived from 20 mL of peripheral blood were processed as follows: PBMCs were resuspended in 0.8 mL of PBS containing 0.5% bovine serum albumin fraction V (BSA, Nacalai Tesque, Inc., Nakagyo-ku, Kyoto, Japan), and 2 mM ethylenediaminetetraacetic acid (EDTA, Nacalai Tesque) in a 15 mL conical tube (AGC Techno Glass, Co., Ltd., Haibara-gun, Shizuoka, Japan). To this suspension, 0.2 mL of anti-human CD3-coated MicroBeads (Miltenyi Biotec, Bergisch Gladbach, North Rhine-Westphalia, Germany) was added. After placing the cells/beads suspension at 4°C for 15 min, 10 mL of PBS/0.5% BSA/2 mM EDTA was added, and the tube was centrifuged at 300 × g and 4°C for 10 min. The supernatant was discarded, and the cells/beads pellet was dispersed by tapping and resuspended in 2 mL of PBS/0.5% BSA/2 mM EDTA. The suspension was loaded onto an LD column (Miltenyi Biotec) attached to a magnet equilibrated with 2 mL of PBS/0.5% BSA/2 mM EDTA. The flow-through was collected, and the column was washed twice with 1 mL of PBS/0.5% BSA/2 mM EDTA. The combined flow-through samples were added to 6 mL of Yssel's medium/10% inactivated human AB serum, then centrifuged at 600 × g and 4°C for 5 min. After aspirating the supernatant, the CD3^{-} PBMC pellets were dispersed and resuspended in Yssel's medium/10% heat-inactivated human AB serum to achieve a cell concentration of 2×10^{6}/mL. The cell suspension was dispensed into wells of a 24-well plate (1.5 mL/well) with the addition of 10^{2} IU/mL of interleukin-2 (Kyowa Pharmaceutical Industries Co., Ltd) and 100 ng/mL of IL-18 (Techno Suzuta, Co., Ltd.). The plate was incubated at 37°C with 5% CO_2 under humidified conditions for 4 days. IL-2/IL-18 were added daily during this period. On day 5, the cell suspensions were combined with an equal volume of Yssel's medium/10% inactivated human AB serum, transferred to a 75 cm^{2} flask (AGC Techno Glass Co., Ltd.), and incubated. From days 6 to 8, the cell suspension was diluted with an equal volume of Yssel's medium/10% inactivated human AB serum and split into two portions. Additionally, 100 IU/mL of IL-2 and 100 ng/mL of IL-18 were added. On day 10, the cell suspensions were transferred to 50 mL conical tubes, centrifuged at 600 × g and 4°C for 5 min, and the culture supernatant was aspirated. The cell pellets were dispersed, resuspended in cryopreservation medium (Takara Bio Inc.) at a concentration of 2×10^{7} cells/mL, and transferred into cryopreservation vials (Thermo Fisher Scientific Inc.). The vials were placed at -80°C overnight and then stored in a liquid nitrogen tank until used.

(2) Flow cytometric analysis. PBMC suspensions, both before and after expansion with IL-2/IL-18, were dispensed into wells of a round-bottom 96-well plate to achieve a cell concentration of 2×10^{5} cells/100 μL. The plate underwent centrifugation at 600 × g and 4ºC for 2 min. Following the removal of supernatants by flipping, the cell pellets were dispersed by vortexing and resuspended in 50 μL of phosphate-buffered saline (PBS)/2% fetal calf serum (FCS). This resuspension contained 3 μL of fluorescein isothiocyanate (FITC)-conjugated anti-CD3 or CD56 mAb and phycoerythrin (PE)-conjugated anti-NKG2D, DNAM-1, CD16, HLA-DQ, or CD86, NKG2A, or CD94 mAb (BioLegend Japan, Bunkyo-ku, Tokyo, Japan), or PD-1 mAb (MBL Co., Ltd., Minato-ku, Tokyo, Japan), followed by RPE-conjugated anti-mouse Ig Ab (FUJIFILM Wako Pure Chem, Corp., Chuo-ku, Osaka, Japan). . After a 15-minute incubation on ice, 200 μL of PBS/2% FCS were added to the wells. The plate was then centrifuged at 600 × g and 4ºC for 2 min, and the supernatants were removed. Following vortexing of the plate, 200 μL of PBS/2% FCS were added to the wells. This process was repeated twice, and the cells were ultimately resuspended in 200 μL of PBS/2% FCS for analysis using a FACS Lyrics flow cytometer (Becton, Dickinson and Company, Franklin Lakes, NJ, USA).

(3) Microscopic analysis. PBMCs, both before and after expansion with PTA/IL-2, were observed under a microscope equipped with a 4 × objective lens and a 10 × eyepiece lens. The images were captured using cellSens software ver. 2.3 (Olympus Corp., Hachioji, Tokyo, Japan).

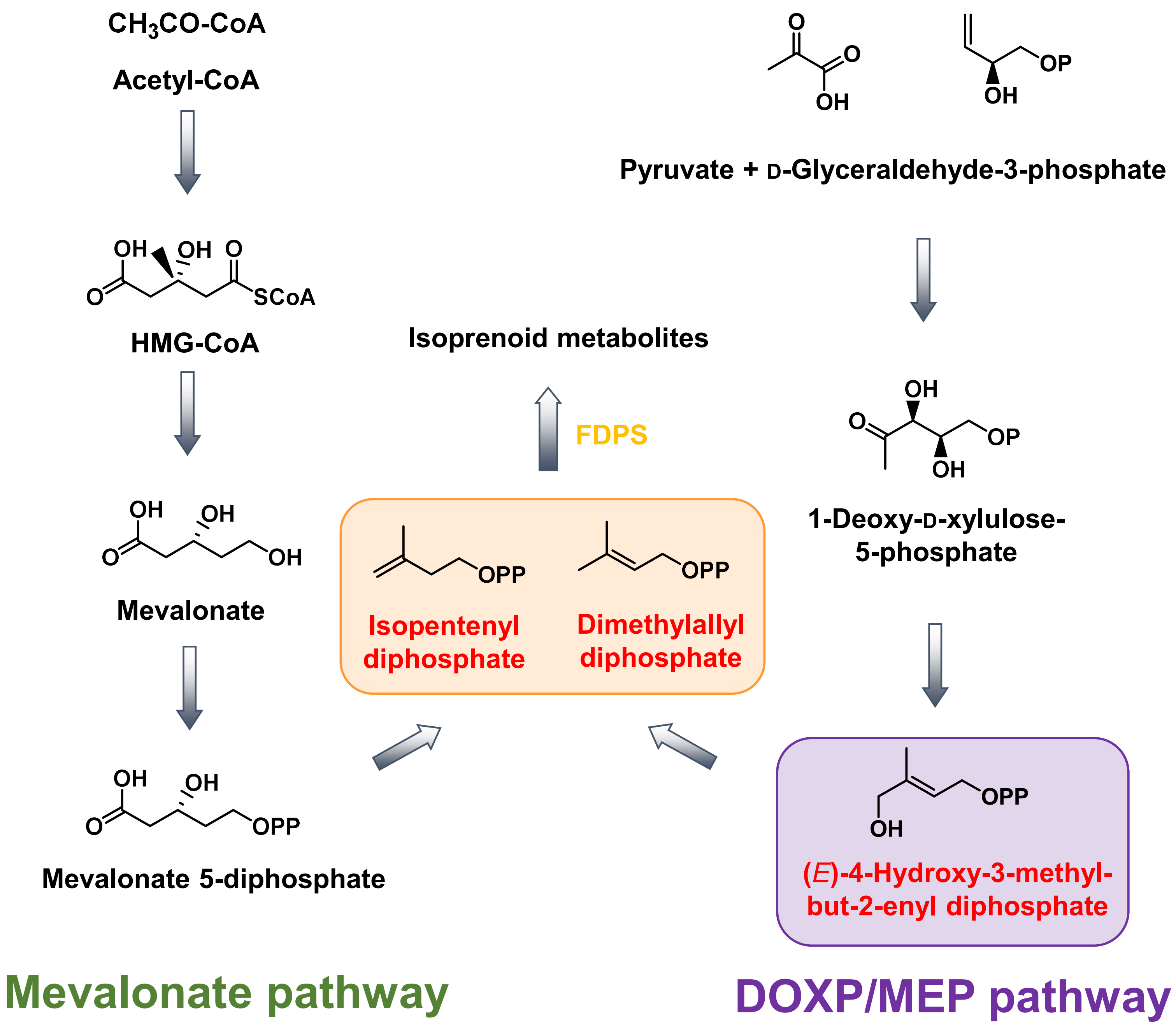

Supplementary Scheme S1. Human and microbial pathways for the biosynthesis of isoprenoid metabolites. In human cells, the process starts with acetyl-CoA, leading to the production of various isoprenoid metabolites including cholesterol, bile acids, steroid hormones, and lipopeptides. Acetyl-CoA is converted to 3-hydroxy-3-methylglutaryl-CoA (HMG-CoA), which then undergoes a series of conversions to yield mevalonate, mevalonate 5-diphosphate, isopentenyl diphosphate (IPP), and dimethylallyl diphosphate (DMAPP). IPP and DMAPP act as antigens for human γδ T cells. Farnesyl diphosphate synthase (FDPS) catalyzes the conversion of IPP and DMAPP into geranyl diphosphate, and subsequently farnesyl diphosphate, from which various isoprenoid metabolites are synthesized. In pathogenic microbes such as *Mycobacterium tuberculosis* and *Plasmodium falciparum*, the biosynthesis begins with pyruvate and **D**-glyceraldehyde-3-phosphate, which are converted into 1-deoxy-**D**-xylulose 5-phosphate (DOXP, also known as 2-*C*-methyl-**D**-erythritol 4-phosphate, MEP), and then into (*E*)-4-hydroxy-3-methylbut-2-enyl diphosphate (HMBPP), and eventually IPP and DMAPP in the non-mevalonate pathway (also known as DOXP/MEP pathway). HMBPP demonstrates stronger activity in stimulating Vδ2 γδ T cells compared to IPP and DMAPP.

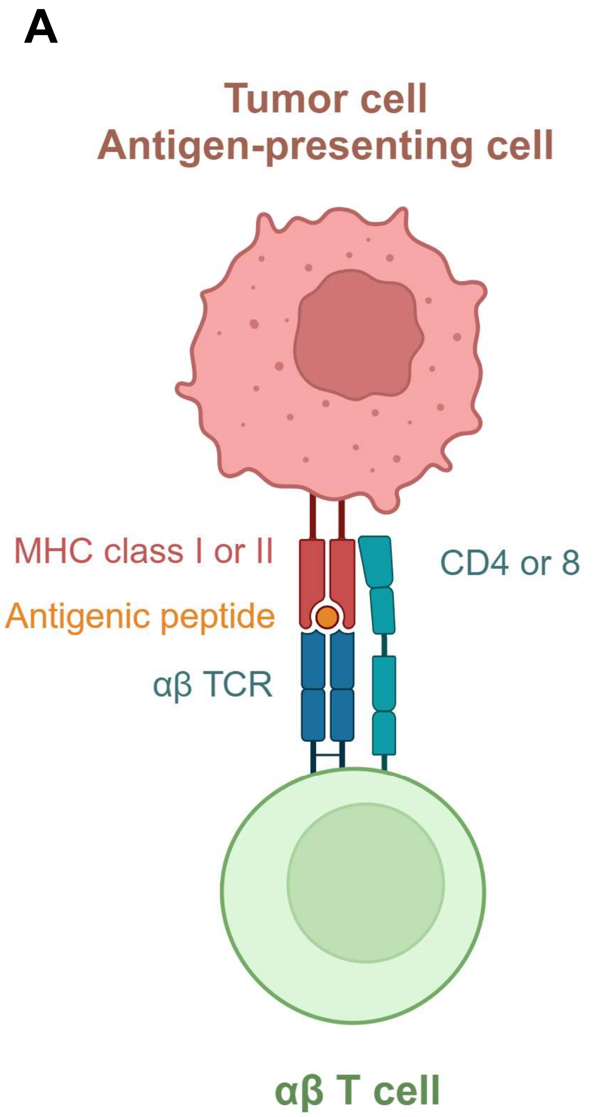

Supplementary Scheme S2. Distinct mechanisms govern the recognition of antigens by αβ T cells and Vδ2 γδ T cells. The majority of αβ T cells recognize antigenic peptides presented by major histocompatibility complex (MHC) class I or II molecules via αβ T cell receptors (TCRs), aided by CD8 or CD4 molecules (A). By contrast, most human Vδ2 γδ T cells expressing Vγ2Vδ2 (also known as V92Vδ2)-bearing TCRs recognize self isopentenyl diphosphate (IPP) and dimethylallyl diphosphate (DMAPP), as well as foreign (*E*)-4-hydroxy-3-methylbut-2-enyl diphosphate (HMBPP). In this recognition process, butyrophilins (BTN) 2A1/3A1 play a crucial role. Upon exposure of human antigen-presenting cells or tumor cells to nitrogen-containing bisphosphonates (N-BPs), these chemicals inhibit farnesyl diphosphate synthase (FDPS), leading to the intracellular accumulation of IPP and DMAPP, direct upstream metabolites of FDPS. IPP and DMAPP then bind to the B30.2 domain of BTN3A1. The interaction between B30.2 and IPP/DMAPP is recognized by Vδ2 γδ T cells in the context of BTN2A1/3A1. (Created with BioRender.com)

Supplementary Table S1. Healthy donors' characteristics.

Healthy donors for functional analysis of Vδ2 γδ T cells

Donor	Age	Sex	Vδ2$^+$ (%)/CD3 on Day 0	Vδ2$^+$ (%)/CD3 on Day11
HD01	29	Female	2.62	99.14
HD02	58	Male	16.17	99.65
HD03	33	Male	13.12	99.72
HD04	31	Male	2.77	98.84
HD05	28	Male	38.44	99.90
HD06	29	Male	2.32	98.47
HD07	22	Male	6.08	99.57

Healthy donors for functional analysis of NK cells

Donor	Age	Sex	CD56$^+$CD3$^-$ (%) on Day 0	CD16$^+$CD56$^+$ (%) on Day10
HD08	35	Male	97.80	94.50
HD09	58	Male	95.00	94.50
HD10	33	Male	95.00	76.90
HD11	29	Female	94.50	90.40
